# The *Staphylococcus aureus* CamS lipoprotein is a repressor of toxin production that shapes host-pathogen interaction

**DOI:** 10.1371/journal.pbio.3002451

**Published:** 2024-01-05

**Authors:** Katrin Schilcher, Morgan M. Severn, Christian Jenul, Young-Saeng C. Avina, Rebecca A. Keogh, Alexander R. Horswill

**Affiliations:** 1 Department of Immunology and Microbiology, School of Medicine, University of Colorado, Anschutz Medical Campus, Aurora, Colorado, United States of America; 2 Department of Genetics and Genome Biology, University of Leicester, Leicester, United Kingdom; 3 Department of Veterans Affairs, Eastern Colorado Health Care System, Aurora, Colorado, United States of America; Brigham and Women’s Hospital, UNITED STATES

## Abstract

Lipoproteins of the opportunistic pathogen *Staphylococcus aureus* play a crucial role in various cellular processes and host interactions. Consisting of a protein and a lipid moiety, they support nutrient acquisition and anchor the protein to the bacterial membrane. Recently, we identified several processed and secreted small linear peptides that derive from the secretion signal sequence of *S*. *aureus* lipoproteins. Here, we show, for the first time, that the protein moiety of the *S*. *aureus* lipoprotein CamS has a biological role that is distinct from its associated linear peptide *staph*-cAM373. The small peptide was shown to be involved in interspecies horizontal gene transfer, the primary mechanism for the dissemination of antibiotic resistance among bacteria. We provide evidence that the CamS protein moiety is a potent repressor of cytotoxins, such as α-toxin and leukocidins. The CamS-mediated suppression of toxin transcription was reflected by altered disease severity in in vivo infection models involving skin and soft tissue, as well as bloodstream infections. Collectively, we have uncovered the role of the protein moiety of the staphylococcal lipoprotein CamS as a previously uncharacterized repressor of *S*. *aureus* toxin production, which consequently regulates virulence and disease outcomes. Notably, the *camS* gene is conserved in *S*. *aureus*, and we also demonstrated the muted transcriptional response of cytotoxins in 2 different *S*. *aureus* lineages. Our findings provide the first evidence of distinct biological functions of the protein moiety and its associated linear peptide for a specific lipoprotein. Therefore, lipoproteins in *S*. *aureus* consist of 3 functional components: a lipid moiety, a protein moiety, and a small linear peptide, with putative different biological roles that might not only determine the outcome of host–pathogen interactions but also drive the acquisition of antibiotic resistance determinants.

## Abstract 2

Staphylococcus aureus lipoproteins fulfill unique biological roles. Katrin Schilcher and co-workers revealed that the staphylococcal lipoprotein CamS represses toxin production. This discovery sheds light on how a lipoprotein contributes to virulence and shapes disease outcomes.

## Introduction

The Gram-positive bacterium *Staphylococcus aureus* is a frequent colonizer of the skin and mucosae of the human host [1,2] and is often the cause of skin and soft tissue infections (SSTIs). However, after gaining access to deeper tissues, *S*. *aureus* can also cause a variety of more severe diseases, including sepsis, endocarditis, pneumoniae, and osteomyelitis [3,4]. In order to survive this versatile lifestyle and changing environmental conditions, *S*. *aureus* needs to sense and react to different environmental cues and regulate a defined set of virulence factors, among which surface proteins play a preeminent role [5]. It is therefore not surprising that the bacterial surface proteome is dominated by proteins that facilitate interaction with the environment, many of which are lipoproteins (Lpp) [6]. Lpp, present in all bacteria, are anchored to bacterial membranes through their N-terminal lipid moiety [7,8]. Most research studies characterize 2 components of bacterial Lpp, the protein and the lipid moiety. Due to their localization at the interface of the extracellular environment and the bacterial surface, the protein moiety of *S*. *aureus* Lpp can have diverse binding and enzymatic activities [9,10], while the lipid part is a ligand for Toll-like receptor 2 (TLR2) and crucial for immune modulation [11–14]. A third component, a small linear peptide derived from the secretion signal sequences of Lpp precursors, has only been studied in a few bacterial species [15–17].

Although several *S*. *aureus* Lpp are associated with transporters and contribute to virulence [18], the vast majority of staphylococcal Lpp have not been investigated. In this study, we characterize the CamS Lpp and show that it is a repressor of toxin production in *S*. *aureus* USA300, the current epidemic methicillin-resistant *S*. *aureus* (MRSA) lineage in the United States [19]. This represents a novel biological role that is distinct from the involvement of its linear peptide *staph*-cAM373 in horizontal gene transfer (HGT) [20]. Using mutational analysis, we demonstrate that CamS is a potent repressor of genes encoding cytotoxins, such as α-toxin and leukocidins (*lukA*, *lukB*, *hlgA*, *hlgB*, *hlgC*). Mutation of the protein component of CamS increases *S*. *aureus* hemolysis of rabbit red blood cells and cytotoxicity towards human polymorphonuclear leukocytes. We show that CamS-mediated gene regulation is critical for *S*. *aureus* pathogenesis and mutation of *camS* leads to a significant increase in virulence in a murine SSTI as well as an in vivo sepsis model. Our discoveries provide evidence for distinct biological functions of an Lpp protein moiety and its associated linear peptide. In addition, the here presented study serves as a starting point for future investigations into the interplay between the newly characterized virulence-associated Lpp CamS with other regulatory systems in *S*. *aureus*.

## Results

### CamS represses *S*. *aureus* virulence factors

**Fig 1:**
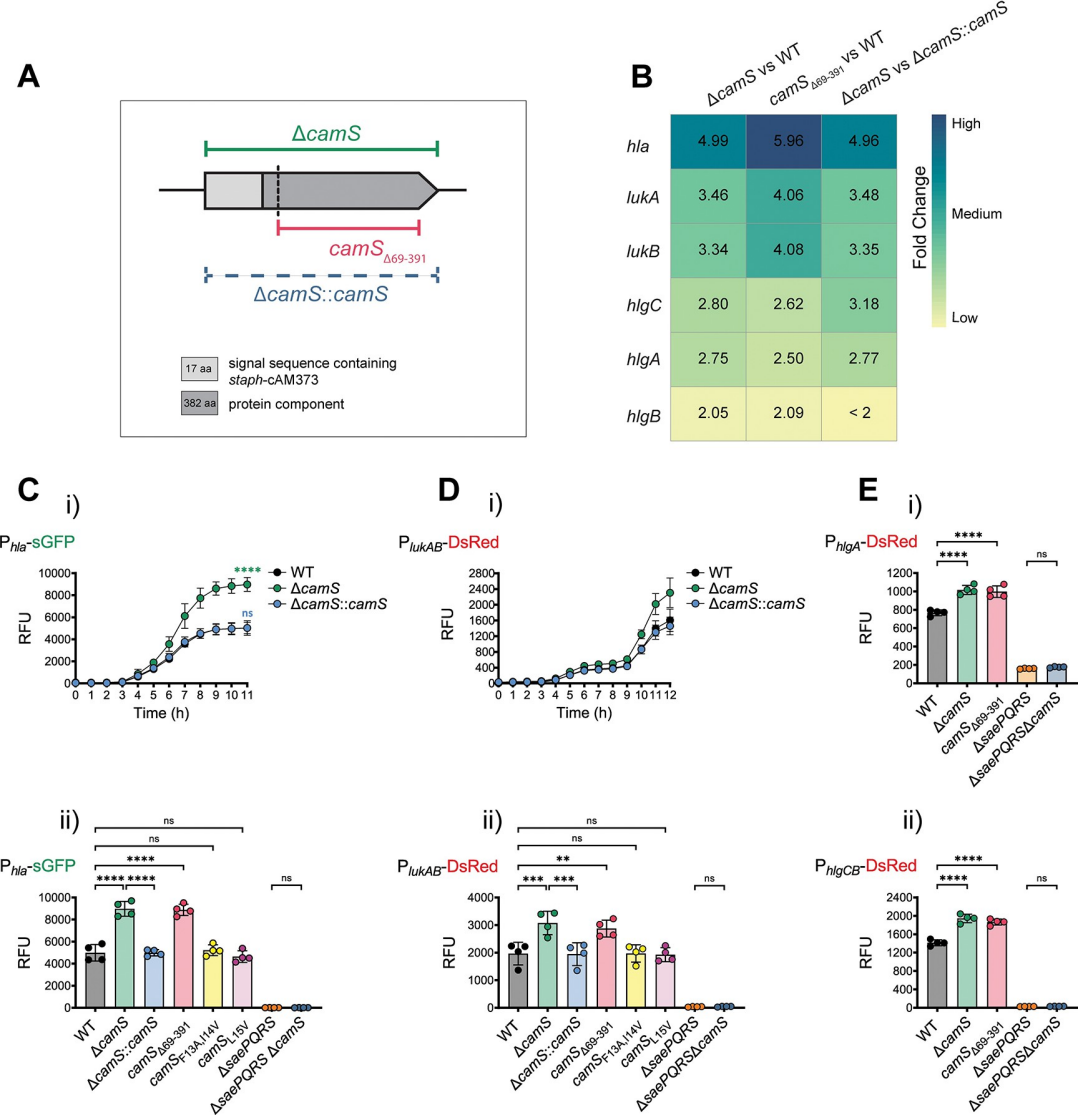
The CamS protein moiety represses *S*. *aureus* toxin expression. (**A)** Schematic of the *S*. *aureus camS* gene, highlighting the region missing in the in-frame *camS* deletion mutant (Δ*camS*), the *camS* truncation mutant that lacks 323 amino acids of the protein moiety but retains the signal sequence containing *staph*-cAM373 (*camS*_Δ69–391_), and the *camS* chromosomal complementation in the Δ*camS* mutant background (Δ*camS*::*camS*). The position used for *camS* truncation is indicated by a dashed vertical line. **(B)** RNA-seq heat map of toxins differentially expressed in Δ*camS* versus WT, *camS*_Δ69–391_ versus WT and Δ*camS* versus Δ*camS*::*camS* (>2-fold change in expression and adjusted *P* value <0.01). **(C)** Quantitative determination of the RFU in **(i)** P_*hla*_-sGFP expressing WT, Δ*camS*, and Δ*camS*::*camS* over the course of 11 h (*n* = 4) and in **(ii)** P_*hla*_-sGFP expressing WT, Δ*camS*, Δ*camS*::*camS*, *camS*_Δ69–391_, *camS*_F13A,I14V,_ *camS*_L15V_, Δ*saePQRS*, and Δ*saePQRS* Δ*camS* double mutant after 24 h (*n* = 4). **(D)** Quantitative determination of the RFU in **(i)** P_*lukAB*_-DsRed-expressing WT, Δ*camS*, and Δ*camS*::*camS* over the course of 12 h (*n* = 3) and in **(ii)** P_*lukAB*_-DsRed-expressing WT, Δ*camS*, Δ*camS*::*camS*, *camS*_Δ69–391_, *camS*_F13A,I14V,_ *camS*_L15V_, Δ*saePQRS*, and Δ*saePQRS* Δ*camS* double mutant after 24 h (*n* = 4). **(E)** Quantitative determination of the RFU in **(i)** P_*hlgA*_-DsRed-expressing WT, Δ*camS*, *camS*_Δ69–391_, Δ*saePQRS*, and Δ*saePQRS* Δ*camS* and **(ii)** P_*hlgCB*_-DsRed-expressing WT, Δ*camS*, *camS*_Δ69–391_, Δ*saePQRS*, and Δ*saePQRS* Δ*camS* after 24 h (*n* = 4). Results represent the pooled data from independent experiments (individual dots), and all data are shown as mean ± SD. Significant differences for Δ*camS* or Δ*camS*::*camS* compared to WT at time point 11 h in (Ci) were determined by one-way ANOVA with Dunnett’s multiple comparison test. Significant differences between the data sets in (Cii), (Dii), (Ei), and (Eii) were determined by one-way ANOVA with Bonferroni’s multiple comparisons test. **P < 0.01, ***P < 0.001, ****P < 0.0001, ns = not significant. The data underlying panel B can be found in S1 Table, and the data underlying panels C, D, and E can be found in S1 Data. RFU, relative fluorescence unit; RNA-seq, RNA-sequencing; WT, wild type.

To delineate the biological role of CamS, we used an in-frame *camS* deletion mutant (Δ*camS*) in the MRSA USA300 wild type (WT) background, a truncated CamS mutant lacking 323 amino acids of the CamS protein moiety (*camS*_Δ69–391_), and a chromosomal complementation of the *camS* mutant (Δ*camS*::*camS*) (**Fig 1A**). The *camS*_Δ69–391_ mutant and the WT produced similar amounts of *staph*-cAM373 (**S1A Fig**), as assessed by a previously described *Enterococcus faecalis* aggregation assay [16]. To determine the transcriptional impact of CamS, we performed RNA-sequencing (RNA-seq) of MRSA USA300 WT, Δ*camS*, *camS*_Δ69–391_, and Δ*camS*::*camS* cultures grown to late-stationary phase in which the predicted *camS* promoter (**S1Bi Fig**) showed high activity (**S1Bii Fig**). Genes that showed 2-fold higher or lower expression compared to WT and had an adjusted *P* value below 0.01 were considered differentially expressed in a CamS-dependent manner. Differential gene expression analysis comparing Δ*camS* or *camS*_Δ69–391_ with the WT showed an altered transcriptional response of several virulence factors (**S1 Table**). Similar results were obtained when comparing the Δ*camS* mutant with Δ*camS*::*camS* (**S1 Table**). Among the genes up-regulated in the *camS* mutants, we detected genes encoding toxins that are associated with staphylococcal virulence and human infection, such as α-toxin (*hla*) and several leukocidins (*lukA*, *lukB*, *hlgA*, *hlgB*, *hlgC*) (**Fig 1B**). While several other genes were also differentially regulated, including genes associated with metabolism and transport, we decided to focus on characterizing the CamS-mediated repression of *S*. *aureus* toxins.

We then validated the regulation of distinct toxins with transcriptional reporters. Construction of promoter fusion plasmids was based on predicted transcription start sites [21] or transcriptomic data from our laboratory. The activity of the *hla* promoter, fused to a green fluorescent protein reporter gene (P_*hla*_-sGFP), was substantially higher in Δ*camS*, compared to the WT and Δ*camS*::*camS*, confirming that CamS represses *hla* (**Fig 1Ci**). To further understand whether the protein moiety or linear peptide moiety of the CamS Lpp was responsible for the observed effect, site-directed mutagenesis was used to exchange 1 or 2 amino acid residues within the *staph*-cAM373 sequence (“AIFILAA”) to produce inactive linear peptides that did not induce *staph*-cAM373-mediated *E*. *faecalis* aggregation (*camS*_F13A,I14V_, *camS*_L15V_) (**S1A Fig**). None of the strains showed differences in bacterial growth under the conditions used (**S1Ci and S1Cii Fig**). In this extended strain set, expression of the P_*hla*_-sGFP fusion was approximately 2-fold stronger after 24 h in the Δ*camS* and *camS*_Δ69–391_ mutants, compared to the WT and Δ*camS*::*camS* (**Fig 1Cii**). In addition, both peptide mutants showed *hla* promoter activities similar to the WT (**Fig 1Cii**), suggesting that the protein moiety of CamS represses *hla*. The *S*. *aureus* exoprotein (Sae) two-component system (TCS), consisting of the SaeR response regulator, its cognate sensor kinase SaeS, and the 2 accessory proteins SaeP and SaeQ [22,23], was shown to activate *hla* transcription by binding to the consensus SaeR-binding site upstream of the *hla* promoter [24,25]. As expected, measuring the *hla* promoter activity in the *saePQRS* whole gene locus mutant (Δ*saePQRS*) confirmed that expression of *hla* is SaeRS dependent. Deletion of *camS* in the Δ*saePQRS* background (Δ*saePQRS* Δ*camS*) showed no significant difference in *hla* promoter activity compared to Δ*saePQRS* (**Fig 1Cii**) demonstrating that *hla* repression by CamS is dependent on its prior activation by SaeRS.

As shown in **Fig 1Di**, the *lukAB* promoter activity (*lukAB* promoter sequence fused to a red fluorescent protein reporter gene; P_*lukAB*_-DsRed) showed a stronger increase over 12 h in Δ*camS* compared to the WT and Δ*camS*::*camS*. We also measured the expression of the P_*lukAB*_-DsRed fusion after 24 h in an extended strain set, demonstrating that expression of *lukAB* was significantly stronger in the Δ*camS* and *camS*_Δ69–391_ backgrounds, compared to the WT and Δ*camS*::*camS* (**Fig 1Dii**). Similar to our *hla* promoter study, the *lukAB* promoter activities in the 2 peptide mutant strains were comparable to the WT (**Fig 1Dii**). Since the transcription of leukocidins is also regulated by SaeRS [24], we measured the *lukAB* promoter activity in a Δ*saePQRS* mutant as well as the Δ*saePQRS* Δ*camS* double mutant. Both mutant strains showed no P_*lukAB*_-DsRed promoter activity after 24 h (**Fig 1Dii**). The γ-hemolysin locus consists of 3 genes, *hlgA* with its own promoter, and the *hlgC* and *hlgB* operon [26]. We measured activity of both promoter fusions, P_*hlgA*_-DsRed and P_*hlgCB*_-DsRed in WT, Δ*camS*, and *camS*_Δ69–391_ as well as in Δ*saePQRS* and the Δ*saePQRS* Δ*camS* double mutant. Similar to promoter activities of *hla* and *lukAB*, the activities of the *hlgA* and *hlgCB* promoters were significantly higher in strains lacking the CamS protein moiety (Δ*camS* and *camS*_Δ69–391_) and very low or undetectable in the Δ*saePQRS* single mutant and the Δ*saePQRS* Δ*camS* double mutant (**Fig 1Ei and 1Eii**).

The *camS* gene is conserved in the genomes of several *S*. *aureus* strains (**S1D Fig**), and similar *camS* expression was observed with a *camS* promoter fusion (P_*camS*_-DsRed) in the USA400 MW2 and USA300 LAC strains (**S1Ei Fig**). Previous in vitro transcription studies showed that USA300 isolates expressed *hla* at much higher levels than USA400 isolates, including strain MW2 [27]. Our promoter fusion results confirm the much lower in vitro *hla* expression in USA400 compared to USA300 (**S1Eii and S1Eiii Fig**). Similar to USA300, deletion of *camS* in the USA400 genetic background leads to increased *hla* promoter activity (P_*hla*_-DsRed) compared to the WT (**S1Eii and S1Eiii Fig**).

Collectively, our in vitro data showed that the disruption of the protein moiety of CamS leads to the derepression of several toxins and suggest that CamS contributes to *S*. *aureus* virulence regulation across lineages.

### CamS-mediated toxin regulation contributes to *S*. *aureus* virulence in vivo

**Fig 2:**
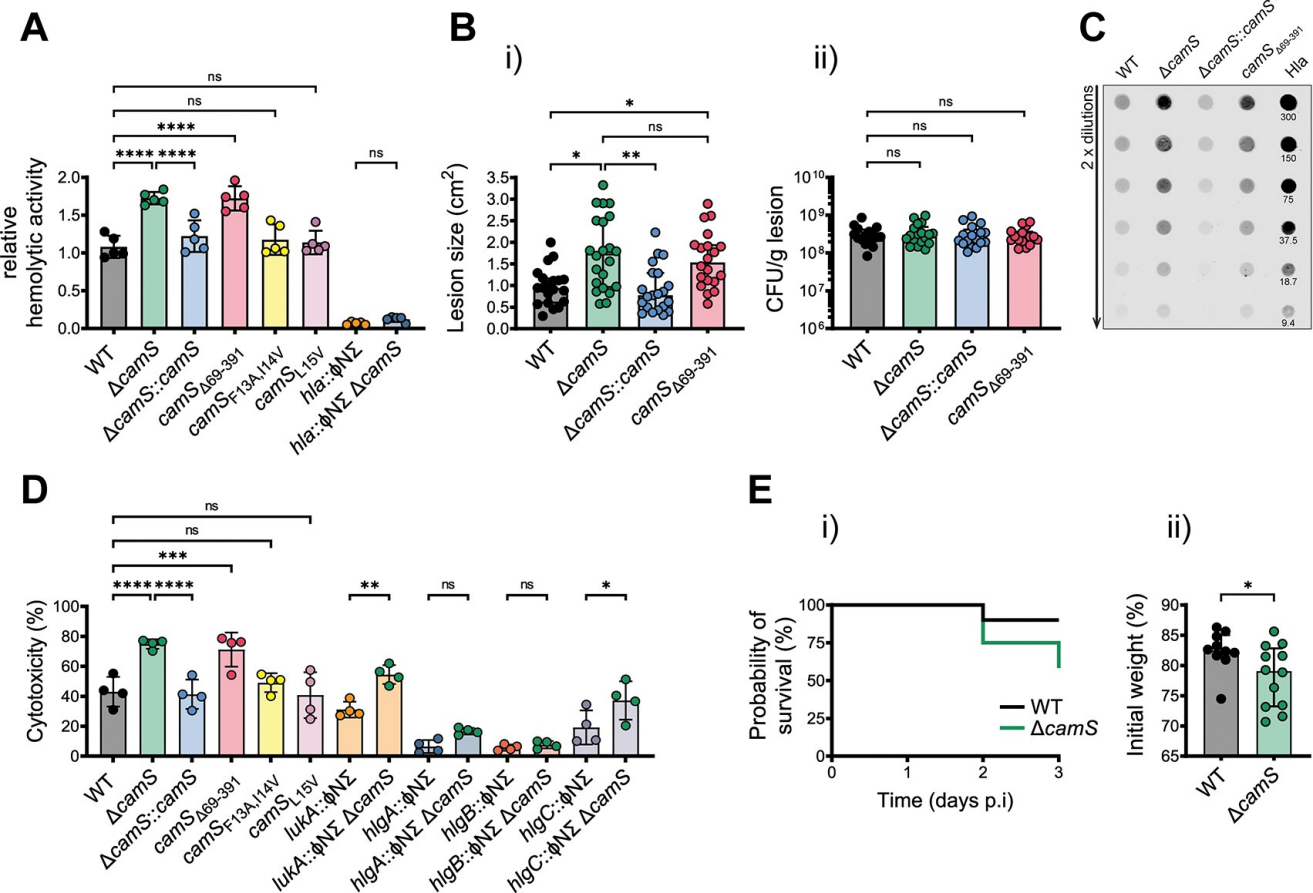
CamS-mediated toxin regulation determines *S*. *aureus* virulence. **(A)** Relative hemolytic activity of culture filtrates from WT, Δ*camS*, Δ*camS*::*camS*, *camS*_Δ69–391_, *camS*_F13A,I14V_, *camS*_L15V_, *hla*::ΦNƩ, and *hla*::ΦNƩ Δ*camS* double mutant towards rabbit RBC (*n* = 5). Results represent the pooled data from independent experiments (individual dots), and all data are shown as mean ± SD. Significant differences were determined by one-way ANOVA with Bonferroni’s multiple comparison test. ****P < 0.0001, ns = not significant. **(Bi)** Dermonecrotic skin lesion size of BALB/cJ mice infected with WT, Δ*camS*, Δ*camS*::*camS*, and *camS*_Δ69–391_ after 6 days postinfection. Data were pooled from 6 independent experiments and presented as median with interquartile range (individual dots = mice, 20 for WT, 22 for Δ*camS*, 21 for Δ*camS*::*camS*, 20 for *camS*_Δ69–391_). Significant differences were determined by a Kruskal–Wallis test followed by a post hoc Dunn’s multiple comparison test. *P < 0.05, **P < 0.01, ns = not significant. **(ii)** Bacterial burden, measured as CFU/gram (CFU/g) of dermonecrotic lesions at 6 days postinfection for the indicated groups. Data were pooled from 5 independent experiments presented as median with interquartile range (individual dots = mice, 16 for WT, 18 for Δ*camS*, 17 for Δ*camS*::*camS*, 16 for *camS*_Δ69–391_). Significant differences were determined by a Kruskal–Wallis test followed by a post hoc Dunn’s multiple comparison test. ns = not significant. **(C)** Dot immunoblot of homogenized tissues from dermonecrotic lesions (day 6) from WT, Δ*camS*, Δ*camS*::*camS*, and *camS*_Δ69–391_ strains. Two-fold dilutions of the homogenates or recombinant Hla protein from *S*. *aureus* (9.4–300 ng) were spotted on a nitrocellulose membrane and probed using Hla antibodies. A representative dot immunoblot from 3 independent experiments is shown. **(D)** Intoxication of hPMN with culture filtrates collected from WT, Δ*camS*, Δ*camS*::*camS*, *camS*_Δ69–391_, *camS*_F13A,I14V_, *camS*_L15V_, *lukA*::ΦNƩ, *lukA*::ΦNƩ Δ*camS*, *hlgA*::ΦNƩ, *hlgA*::ΦNƩ Δ*camS*, *hlgB*::ΦNƩ, *hlgB*::ΦNƩ Δ*camS*, *hlgC*::ΦNƩ, and *hlgC*::ΦNƩ Δ*camS*. Cytotoxicity towards hPMN was measured via LDH release (*n* = 4). Results represent the pooled data from independent experiments (individual dots), and all data are shown as mean ± SD. Significant differences were determined by one-way ANOVA with Bonferroni’s multiple comparison test. *P < 0.05, **P < 0.01, ***P < 0.001, ****P < 0.0001, ns = not significant. **(E)** In vivo virulence of WT and Δ*camS* in a murine sepsis model (C57BL/6J). **(i)** The probability of survival over 3 days and **(ii)** the percent weight loss at 2 days postinfection are reported and presented as median with interquartile range. Results represent the pooled data from 2 independent experiments (individual dots = mice, 10 for WT, 12 for Δ*camS*). Significant differences were determined by a Mann–Whitney *U* test, *P < 0.05. The data underlying panels A, B, D, and E can be found in S1 Data. CFU, colony-forming unit; hPMN, human polymorphonuclear leukocytes; LDH, lactate dehydrogenase; RBC, red blood cells; WT, wild type.

Consistent with up-regulation of *hla* in strains lacking the *camS* protein moiety (**Fig 1Cii**), we detected elevated hemolysis of rabbit red blood cells (RBC) in the presence of stationary-phase culture filtrates from Δ*camS* and *camS*_Δ69–391_ relative to the WT strain (**Fig 2A and S2 Table**). Comparable to our *hla* promoter activity studies, Δ*camS*::*camS* and both peptide mutant strains (*camS*_F13A,I14V_ and *camS*_L15V_) showed hemolytic activity similar to the MRSA USA300 WT. Culture filtrates of the *hla* mutant (*hla*::ΦNƩ) revealed that α-toxin is essential for the observed RBC lysis under the tested conditions and simultaneous inactivation of *hla* and *camS* (*hla*::ΦNƩ Δ*camS*) phenocopied the *hla* single mutant (**Fig 2A**).

Based on our in vitro data, we hypothesized that mutation of the CamS protein moiety would translate to increased *S*. *aureus* virulence in vivo. To determine the role of CamS in murine skin infection, animals were subcutaneously injected with MRSA USA300 WT and mutant strains. Similar to the *hla* mutant strain (*hla*::ΦNƩ), the *hla*::ΦNƩ Δ*camS* double mutant lacked the capacity to form skin lesions in our murine SSTI model (**S2Ai–S2Aiii Fig**), confirming the predominant role of α-toxin to establish cutaneous infections [28,29]. At day 6 postinfection, no significant difference in weight loss was observed in mice infected with any of the 4 strains (**S2Aiv Fig**). However, mice infected with Δ*camS* and *camS*_Δ69–391_ showed significantly higher lesion sizes compared to the WT or Δ*camS*::*camS*, suggesting that the protein moiety of CamS also represses *hla* expression in vivo (**Fig 2Bi**). To investigate whether the more severe skin lesions were associated with increased bacterial burden in the local skin tissue, skin biopsies were collected on day 6 postinfection and homogenized to enumerate colony-forming units (CFU counts per gram tissue). No significant difference in the bacterial load was observed comparing the 4 strains (**Fig 2Bii**). We hypothesized that the observed increase in lesion size produced by Δ*camS* and *camS*_Δ69–391_ is likely due to higher α-toxin production. Subsequently, we examined the amount of α-toxin (Hla) in tissue homogenates at day 6 by dot immunoblot (**Figs 2C and S2B**). A substantial increase of Hla was detected in tissue homogenates of mice infected with Δ*camS* and *camS*_Δ69–391_ compared to WT and Δ*camS*::*camS*. Based on our results, we conclude that the CamS protein component acts as a repressor of α-toxin production in our murine SSTI model.

Humans are more sensitive to a wider range of *S*. *aureus* toxins than mice [30,31]. To gain a deeper understanding of the extent of CamS-mediated virulence regulation, we also investigated *S*. *aureus*-mediated killing of human polymorphonuclear leukocytes (hPMN), representing the first line of defense against *S*. *aureus* infections [32]. To evaluate the contribution of each up-regulated leukocidin (**Fig 1B**) to hPMN killing, culture filtrates collected from 24-h grown WT and mutant strains were used to intoxicate hPMN. Killing of hPMN was assessed by the amount of cytoplasmic lactate dehydrogenase (LDH) released into the culture supernatant. As shown in **Fig 2D**, consistent with the previously observed increased expression of secreted leukocidins in Δ*camS* and *camS*_Δ69–391_ (**Fig 1D and 1E**), culture filtrates of both mutant strains were significantly more cytotoxic towards hPMN than culture filtrates from WT, Δ*camS*::*camS*, and the 2 peptide mutants (*camS*_F13A,I14V_ and *camS*_L15V_). Incubation of hPMN with culture filtrates of the isogenic *lukA* transposon mutant (*lukA*::ϕNƩ) only showed a slight reduction in cytotoxicity compared to the WT strain. However, culture filtrates of transposon mutants in the γ-hemolysins *hlgA* and *hlgB* resulted in nearly complete abolishment of cytotoxicity, while mutation of *hlgC* still showed residual cytotoxicity (**Fig 2D**). To further demonstrate that the observed hypercytotoxic phenotype was due to the inactivation of CamS, a *camS* deletion was introduced into the leukocidin transposon mutants (*lukA*, *hlgA*, *hlgB*, *hlgC*). Mutation of *camS* in the *lukA* and *hlgC* mutant backgrounds resulted in a significant increase in cytotoxicity compared to the *lukA* and *hlgC* single mutant strain, respectively. No significant increase in cytotoxicity was observed upon introduction of the *camS* mutation in the *hlgA* and *hlgB* mutant backgrounds, demonstrating that CamS-driven repression mainly affects HlgAB-mediated cytotoxicity in our hPMN killing experiment.

Although *S*. *aureus* predominantly results in SSTI, local infections allow access to deeper tissues from where *S*. *aureus* enters the bloodstream, leading to bacteremia and the development of sepsis [33]. Recently, it was shown that leukocyte–bacteria interactions determine sepsis outcome [34], and we hypothesized that CamS-mediated toxin repression might impact progression and outcome of sepsis. The virulence of the MRSA USA300 WT and the Δ*camS* mutant were compared in a 3-day murine sepsis model of infection. After mice were inoculated with equal amounts of the 2 strains, virulence was assessed by monitoring murine survival and weight loss of the infected animals. Mice infected with Δ*camS* showed a trend of decreased survival over the course of the experiment (**Fig 2Ei**). At day 2 postinfection, mice infected with Δ*camS* displayed a significant weight loss compared to the mice infected with the WT (**Fig 2Eii**). No differential colonization of organs (liver, kidney) could be detected between the 2 strains (**S2Ci and S2Cii Fig**). The increased mortality rate and weight loss in animals infected with Δ*camS* suggests that the up-regulation of toxins in this mutant leads to increased virulence in our murine sepsis model.

## Discussion

Despite a wealth of knowledge on virulence factor regulation in *S*. *aureus*, we are just beginning to explore the regulatory networks involving Lpp components in *S*. *aureus*. Recently, we identified the processing and secretion machinery for Lpp-derived linear peptides in *S*. *aureus* [16]. The small linear peptide *staph*-cAM373, derived from the secretion signal sequence of the Lpp encoded by *camS*, shares homology with a linear peptide in *E*. *faecalis* that regulates conjugal plasmid transfer [20]. While *staph*-cAM373 was shown to induce interspecies HGT between *E*. *faecalis* and *S*. *aureus* in vitro [20], the 43-kDa CamS protein moiety has not been studied yet. By combining transcriptomics, bacterial genetics, functional in vitro assays and in vivo studies, we demonstrate that the CamS protein moiety acts as a repressor of α-toxin (*hla*) and several leukocidins (*lukA*, *lukB*, *hlgA*, *hlgB*, *hlgC*). The loci encoding the CamS-regulated toxins are part of the *S*. *aureus* core genome and are present in approximately 99% of sequenced *S*. *aureus* strains [35–38], suggesting that CamS-mediated virulence suppression is likely conserved in the species *S*. *aureus*. Indeed, we showed that the deletion of *camS* in another *S*. *aureus* lineage, USA400, also results in the transcriptional up-regulation of α-toxin. The abovementioned toxins are all under the regulatory control of the Sae TCS in *S*. *aureus* [24,25] and mutation of *sae* leads to the loss of observable toxin repression by CamS. Some bacterial Lpp were shown to be an integral part of TCSs and/or interact with kinases and thereby modulate their regulatory activity [23,39,40]. A mechanism that might also underlie CamS-mediated transcriptional repression of toxins.

Although α-toxin is capable of lysing various cell types, rabbit erythrocytes exhibit greater sensitivity to α-toxin exposure compared to human erythrocytes, attributed to the absence of the high-affinity toxin receptor ADAM10 (A Disintegrin and Metalloproteinase 10) on human RBC [41]. Accordingly, we used rabbit RBC in our experimental setup to capture the full extent of CamS-mediated α-toxin repression. Our findings indicate that the up-regulation of α-toxin, mediated by the deletion of the CamS protein moiety, results in enhanced lysis of rabbit RBC. We also addressed herein the role of CamS-mediated killing of hPMN. MRSA USA300 can produce up to 5 different pore-forming, bicomponent leukocidins (γ-hemolysins HlgAB and HlgCB, LukSF-PV/PVL, LukAB/HG, and LukED) with the ability to lyse hPMN [38,42]. We did not observe differential expression of the leukocidins *lukSF-PV* and *lukED* in our RNA-seq analysis, but culture broth-dependent expression of both leukocidins were reported in MRSA USA300 [31]. A *lukA* mutant strain only showed a slight reduction in cytotoxicity towards hPMN compared to the WT strain, despite *lukA* and *lukB* being the 2 most highly CamS-repressed leukocidins in our transcriptomics data. This discrepancy is most likely explained by previous findings, demonstrating that LukAB is primarily associated with the bacterial cell during stationary phase when grown in rich media [43,44]. Therefore, despite the elevated expression and promoter activity of *lukAB* in the *camS* mutant strain, other secreted leukocidins might have a stronger impact on hPMN cytotoxicity under our experimental conditions. Indeed, deletion of the γ-hemolysins *hlgA*, *hlgB*, or *hlgC* resulted in minimal cytotoxic activity towards hPMN. Based on our results and the bicomponent nature of HlgAB and HlgCB, the increased cytotoxicity of the *camS* mutant strain seems to be mainly driven by derepression of the HlgAB toxin under our experimental conditions.

Our in vitro data on CamS-mediated toxin repression were reflected in two different in vivo models of infection. MRSA USA300 is the predominant cause of SSTIs [45] with α-toxin contributing to superficial and invasive disease and disturbing host immunity during skin infections and recurring disease [46,47]. By using *camS* mutant strains, either lacking the CamS protein moiety, a functional *staph*-cAM373 peptide or both, we demonstrate that the absence of the CamS protein moiety, but not *staph*-cAM373, leads to significantly increased lesion size in our infection model. We also tested the importance of CamS in a sepsis model of infection, and we observed a clear trend in increased mortality and weight loss in animals infected with a *camS* mutant strain. It was previously shown, that γ-hemolysins contribute to virulence during systemic infection [48] and septic arthritis [49]. However, murine neutrophils lack the responsive receptors for HlgAB and HlgCB [31], and the impact of CamS-mediated leukocidin repression might not be fully captured in our murine sepsis model. Future research involving a systemic infection model in rabbits, which are more sensitive to infection by *S*. *aureus*, is needed to provide detailed insights into CamS-mediated virulence regulation in bacteremia and sepsis.

Our results show that the linear peptide and the protein moiety of a *S*. *aureus* Lpp can have distinct biological functions. With widespread *camS* representation in staphylococcal genomes, further studies will show whether CamS has additional roles in different *S*. *aureus* strains and lineages. Our RNA-seq analysis revealed that CamS regulates not only genes encoding toxins but also other important virulence factors, including the serine protease-like (Spl) proteases. It remains to be seen whether CamS interacts with components of known global virulence regulatory systems to fine-tune *S*. *aureus* virulence and host–pathogen interaction.

## Materials and methods

### Ethics statement

All animal work was approved by and performed in accordance with the Institutional Animal Care and Use Committee (IACUC) of the University of Colorado Anschutz Medical Campus under protocol number #00486 and #1137.

### Bacterial strains and plasmids

Bacterial strains and plasmids are listed in **S3 Table,** and primers are listed in **S4 Table**. DNA sequencing of the constructed plasmids was performed at the Molecular Biology Service Center at the University of Colorado Anschutz Medical Campus. If not specified otherwise, *S*. *aureus* and *E*. *coli* were cultured in tryptic soy broth (TSB) at 37°C with shaking at 250 rpm. Where appropriate, antibiotics were added to the media at the following final concentrations: chloramphenicol (Cm), 10 μg mL^−1^; erythromycin (Erm), 5 μg mL^−1^. *E*. *coli* strains with plasmids were maintained in media supplemented with ampicillin (Amp) at 100 μg mL^−1^.

### Mice

Seven-week-old male and female BALB/cJ mice (Jackson Laboratories, RRID: IMSR_JAX:000651) and 7-week-old female C57BL/6J mice (Jackson Laboratories, RRID:IMSR_JAX:000664) were purchased from the Jackson Laboratories and were housed in specific pathogen-free facilities at the University of Colorado Anschutz Medial Center Animal Facility. Mice were allowed to acclimate for 1 week prior to experimentation. At experimental endpoints, mice were killed via CO_2_ inhalation followed by cervical dislocation.

### Human polymorphonuclear leukocytes (hPMN)

Human adult polymorphonuclear leukocytes from healthy blood donors were purchased from the Division of Pulmonary, Critical Care and Sleep Medicine at the National Jewish Health at Denver, where they were isolated by the plasma-Percoll method [50].

### Construction of transposon mutants, gene deletions, chromosomal complementation, and *staph*-cAM373 peptide mutants

PCR products were purified with either the QIAquick PCR Purification Kit (QIAGEN; Cat#28106) or the QIAquick Gel Extraction Kit (QIAGEN, Cat#28706). All plasmid purifications were performed with the QIAprep Spin Miniprep Kit (QIAGEN, Cat#27106). Bacteriophage transductions between *S*. *aureus* strains were performed with phage 11 as described previously [51]. All mariner-based transposon *bursa aurealis* mutations from the Nebraska Transposon library [52] (ΦNƩ) were confirmed by PCR with the following primers: KAS284/KAS285 for *hla*::ɸNΣ; KAS408/KAS409 for *lukA*::ɸNΣ; KAS432/KAS433 for *hlgA*::ɸNΣ; KAS434/KAS435 for *hlgB*::ɸNΣ; KAS436/KAS437 for *hlgC*::ɸNΣ.

Genomic DNA of *S*. *aureus* strains was isolated using the Puregene cell kit (QIAGEN; Cat#158567), including a lysis step with lysostaphin (100 ng/μL, ABMI Products LLC, Cat#LSPN). A marker less deletion of *camS* in MRSA USA400 MW2 (MW1844) was constructed as previously described for deleting *camS* (SAUSA300_1884) in the MRSA USA300 background (LAC* Δ*camS*) [16]. Briefly, regions flanking the gene were amplified from MRSA USA400 MW2 genomic DNA (AH843) using primers KAS26/KAS27 and KAS28/KAS29. The amplified products were column purified, digested with *EcoRI*/*XhoI* and *XhoI*/*SalI* (New England Biolabs) and ligated into pJB38, digested with *EcoRI* and *SalI*, to generate pKAS57. The plasmid was electroporated into *E*. *coli* DC10B, sequenced (primers KAS169, KAS170, KAS41, KAS42) and subsequently electroporated into MRSA USA400 MW2, as previously described [53,54]. Deletions were generated as described in [55], and mutants were confirmed by PCR with primers KAS33/KAS34. The previously constructed plasmid pKAS07 (pJB38 with approximately 1,000 bp regions flanking the *camS* gene) [16] was used to introduce a *camS* deletion into LAC* Δ*saePQRS*. The deletion was generated as previously described [55] and confirmed by PCR with primers KAS33/KAS34. Double mutants in *camS* and leukocidins were generated by introducing leukocidin transposon mutations into the Δ*camS* background using bacteriophage transduction as described above.

The *camS* mutation in the LAC* background (LAC* Δ*camS*) was complemented by introducing the *camS* gene at its original location on the chromosome. The *camS* complementation strain (LAC* Δ*camS*::*camS*) was constructed by amplifying the *camS* gene and flanking regions from the MRSA USA300 LAC* genome (AH1263). First, the *camS* gene and a region covering approximately 600 bp upstream of *camS* were amplified with primers KAS266 and KAS281, column purified, digested with *SalI*/*EcoRI* and ligated into pJB38 digested with the same restriction enzymes. The resulting plasmid was electroporated into *E*. *coli* DC10B. After propagation, the plasmid was digested with *XhoI*/*EcoRI* and ligated with a DNA fragment located downstream of the *camS* gene, which was amplified with primers KAS268 and KAS269 and digested with *XhoI*/*EcoRI* to generate pKAS61. The resulting complementation construct carries an *XhoI* restriction site located 43 nt downstream of the *camS* stop codon that serves as a watermark for the complemented strain. Introduction of pKAS61 into *E*. *coli* DC10B, transfer to LAC* Δ*camS*, selection for complemented strains, and plasmid sequencing was carried out as described for construction of the *camS* mutant in MRSA USA400 MW2.

A *camS* mutant strain with a truncated *camS* gene was constructed (LAC* *camS*_Δ69–391_). The expressed truncated CamS protein carries an intact signal peptide sequence that encodes for the *staph*-cAM373 linear peptide but lacks 323 amino acids (aa) of the protein moiety of CamS. Briefly, DNA fragments (approximately 1,000 bp in size) flanking the region targeted for deletion (nt 205 to nt 1,173 of the *camS* gene) were amplified from MRSA USA300 LAC* genomic DNA (AH1263) using primers KAS270/KAS282 and KAS283/KAS272. The product amplified with primers KAS270/KAS282 was digested with *EcoR*/*SalI* and ligated into pJB38 digested with the same enzymes. The resulting plasmid was transferred into *E*. *coli* DC10B by electroporation. After propagation, the plasmid was digested with *SalI*/*NheI* and ligated with the DNA fragment amplified with primers KAS283/KAS272 and digested with the same enzymes to generate pKAS63. Introduction of the resulting plasmids into *E*. *coli* DC10B, transfer to LAC* WT, selection for mutated clones, and plasmid sequencing was carried out as described for construction of the *camS* mutant in MRSA USA400 MW2.

The allelic exchange plasmid pJB38 carrying *camS* was utilized as template to exchange amino acids within the *staph*-cAM373 peptide sequence. In brief, a DNA fragment covering the *camS* gene was PCR amplified from MRSA USA300 LAC* genomic DNA (AH1263) using primers KAS192 and KAS193, digested with restriction enzymes *EcoRI* and *SalI* and ligated into pJB38, resulting in plasmid pKAS38. This plasmid was transferred into *E*. *coli* Top10 by electroporation and sequenced with primers KAS169/KAS170. Site-directed mutagenesis was performed using the QuickChange II Site-Directed Mutagenesis Kit (Agilent Technologies, Cat#200523) with the modified method as recommended by Liu and Naismith [56] with primers KAS194/KAS195 and KAS274/KAS275, resulting in plasmids pKAS39 and pKAS53, respectively. Plasmid pKAS39 has a double nucleotide exchange in a phenylalanine and isoleucine codon to an alanine and valine codon, respectively (F13A/I14V). The plasmid pKAS53 has a single valine substitution in place of a leucine (L15V) in the *staph*-cAM373 peptide amino acid sequence. The mutated plasmids were transferred into *E*. *coli* DC10B by electroporation and sequenced with the primer KAS44. Both plasmids were then electroporated into MRSA USA300 LAC* WT (AH1263) and mutations in the chromosome were generated as described in [55]. Mutations in the *S*. *aureus* genome in both strains LAC* *camS*_F13A,I14V_ and LAC* *camS*_L15V_ were verified by sequencing the amplified PCR product (KAS26/KAS29) using primers KAS175, KAS83, KAS28, KAS44, KAS65, KAS192, and KAS193 from the mutants genome. Numbering corresponds to CamS amino acid sequence.

### Construction of promoter fusion plasmids

Construction of promoter fusion plasmids was based on predicted promoter sequences from Prados and colleagues [21] or promoter predictions using RNA-seq data from [57] and this study. The *hla* promoter-sGFP transcriptional reporter (P_*hla*_-sGFP) was generated by amplification of the putative promoter region of *hla* (SAUSA300_1058) from MRSA USA300 LAC* genomic DNA (AH1263) using primers CLM429/CLM430, digested with *HindIII* and *KpnI* before ligation in the shuttle vector pCM11. The *hla* promoter region fragment fused to sGFP was amplified from the vector with primers CLM463/CLM332, digested with *NheI* and *EcoRI*, and ligated into vector pCM28, resulting in pCM36. An *hla* promoter-DsRed transcriptional reporter (P_*hla*_-DsRed) in MRSA USA300 LAC* and MRSA USA400 MW2 was generated in the shuttle vector pHC48, which expresses DsRed under the control of the *S*. *aureus sarA* P1 promoter [58]. A fragment containing the putative promoter of *hla* was amplified from MRSA USA300 LAC* (AH1263) and USA400 MW2 (AH843) genomic DNA using primers KAS450/KAS451, digested with *XbaI* and *KpnI*, and subsequently ligated upstream of the DsRed gene in pHC48, where the *sarA* promoter fragment was excised using the same enzymes resulting in the reporter plasmid pKAS108 for MRSA USA300 and pKAS109 for MRSA USA400. A *camS* promoter-DsRed transcriptional reporter (P_*camS*_-DsRed) was generated in the shuttle vector pHC48 as described for the *hla* promoter fusion. A fragment containing the *camS* promoter was amplified from MRSA USA300 LAC* genomic DNA (AH1263) using primers KAS175 and KAS193, digested with *SalI* and *KpnI*, and subsequently ligated upstream of the DsRed gene in pHC48, where the *sarA* promoter fragment was excised using the same enzymes resulting in the reporter plasmid pKAS44. Transcriptional reporters for *lukA* (P_*lukA*_-DsRed), *hlgA* (P_*hlgA*_-DsRed), and *hlgCB* (P_*hlgCB*_-DsRed) were constructed in a similar fashion with primers KAS418/KAS419 (P_*lukA*_), KAS446/KAS447 (P_*hlgA*_), and KAS448/KAS449 (P_*hlgCB*_) resulting in the reporter plasmids pKAS92, pKAS103, and pKAS105, respectively. A control strain harboring pHC48 with a promoterless DsRed gene was constructed by excising the *sarA* promoter with *BamHI* and religation of the plasmid (pKAS43). All transcriptional fusion plasmids were transferred into *E*. *coli* DC10B by electroporation [54], sequenced with primers KAS113 and KAS116, and subsequently electroporated into MRSA USA300 LAC* WT, MRSA USA400 MW2 WT, and their respective mutant derivatives.

### Bacterial growth kinetics and yields

To assess growth kinetics of *S*. *aureus* WT and mutant strains, 200 μL of diluted cultures with a starting optical density at 600 nm (OD_600_) of 0.02 in TSB were grown in a clear 96-well microplate and absorbance was measured every 30 min with a Synergy H1 Microplate Reader (BioTek) for 24 h (37°C, continuous orbital shaking). Bacterial growth yields were assessed by counting CFU on tryptic soy agar (TSA) from cultures grown for 24 h at 37°C with agitation at 250 rpm.

### Aggregation assay

To assess the production of the linear peptide *staph*-cAM373 in *S*. *aureus* culture filtrates, we employed a previously reported cell aggregation assay [16,59]. Briefly, *E*. *faecalis* JH2-2 cells containing a peptide-responsive pAM373::Tn*918* plasmid were grown overnight in TSB at 37°C with 10 μg/mL tetracycline at 250 rpm shaking, washed twice, and resuspended to an OD_600_ of 0.2 in chemically defined medium (CDM) [60]. Culture filtrates from *S*. *aureus* WT and mutant strains, grown in CDM until reaching an OD_600_ of 3, were mixed in a 1:1 ratio with *E*. *faecalis* cells in a round-bottom glass tube and incubated at 37°C in a shaking incubator (250 rpm) for 4 ½ h. Cell aggregation was quantified by measuring the turbidity of the suspension with a MicroScan turbidity meter compared to that of the medium.

### Reporter assays

To assess promoter activity, overnight cultures grown with the appropriate antibiotic (37°C, 250 rpm shaking) were washed twice in phosphate-buffered saline (PBS), diluted to an OD_600_ of 0.02 in TSB, and 200 μl were added in triplicate per strain to a 96-well black microplate with clear bottom (Corning Costar, Cat#3603). Plates were incubated at 37°C with shaking (1,000 rpm) in a humidified microtiter plate shaker (Stuart SI505, Cole-Parmer) and absorbance (A_600_) and fluorescence intensity for sGFP (Excitation 480 nm, Emission 515 nm) or DsRed (Excitation 549 nm, Emission 588 nm) were measured with a Tecan Infinite M Plex plate reader. For each experiment, fluorescence values from triplicate wells were averaged, set relative to bacterial growth (A_600_), and the background fluorescence from strains carrying the respective promoterless plasmid (pCM28 or pKAS43) was subtracted. Data are represented as relative fluorescence unit (RFU).

### Hemolysis assay

A rabbit RBC hemolysis assay was used to determine hemolytic activity in bacterial culture filtrates from WT and mutant strains as previously described [61]. Briefly, culture filtrates were generated from cultures grown in TSB for 24 h at 37°C with shaking at 250 rpm. RBC from defibrinated rabbit blood (Hemostat Laboratories, Cat#10052–762) were prepared by washing 4 to 5 times with 1.2 × PBS and resuspending the pellet in 1.2 × PBS at 3% (v/v). Bacterial culture filtrates were serially diluted in 2-fold steps in 1.2 × PBS in a 96-well microtiter plate, mixed with the 3% RBC solution (70:30, RBC:culture filtrates) and incubated statically at room temperature (RT) for 1 ½ hours. Hemolysis was assessed by the loss of turbidity measured at OD_633nm_ using a Tecan Infinite M Plex plate reader. To determine the final concentration (%) of the culture filtrate required for 50% of RBC lysis (EC_50_), the data were analyzed with GraphPad Prism 9 (version 9.4.1.) (4-parameter logistic curve with least squares regression fit). Data are presented as hemolytic activity relative to the median of the WT strain.

### Cytotoxicity assay

A modified Giemsa staining kit (Differential Quik III Stain Kit, Electron Microscopy Sciences, Cat#26096) was used to assess the purity of hPMN (>98%) before each assay. For hPMN infection assays, bacterial cultures grown overnight in TSB (37°C, 250 rpm shaking) were subcultured at a starting OD_600_ of 0.02 and grown for 24 h as described above. All bacterial cultures were normalized to the same OD_600_ and pelleted by centrifugation at 5,000 × *g* for 10 min. Supernatants containing exoproteins were collected, filtered using Costar Spin-X 0.2-μm centrifuge tube filters (Sigma-Aldrich, Cat#CLS8160) and stored at −20°C until usage. *S*. *aureus* culture filtrates (10%, v/v) were used to intoxicate hPMN, seeded at 1 × 10^5^ cells/well in 100 μl RPMI-1640 (Gibco, Cat#11835030) with 10% heat-inactivated fetal bovine serum (FBS; Atlanta Biologicals, Cat#S11550) and incubated for 1 h at 37°C and 5% CO_2_. Following incubation, cells were centrifuged at 300 × *g* for 10 min, and LDH release was assayed as a measure of hPMN viability using the CyQuant LDH Cytotoxicity Assay (Thermo Fisher, Cat#C20300) according to the manufacturer’s instructions. Briefly, 50 μl of cell supernatant was removed and mixed with 50 μl of LDH reagent and incubated for 30 min at RT. *S*. *aureus* growth medium (TSB) was used as 100% viability control, and lysis buffer from the CyQuant LDH Cytotoxicity Assay served as control for 100% cell lysis. Bacterial LDH production in culture filtrates without addition of hPMN was assessed, and no bacterial LDH was detectable. Viability of hPMN was measured with a Tecan Infinite M Plex plate reader (Absorbance at 490 nm and 690 nm). Percentage of cytotoxicity was calculated by subtracting background values (background signal from instrument, growth medium) and normalizing to 100% lysis.

### Sample preparation for RNA purification

Bacterial overnight cultures (37°C, TSB, 250 rpm) were diluted to an OD_600_ of 0.02 in 5 mL TSB, grown for 24 h at 37°C with shaking (250 rpm) and 2 mL of each culture were centrifuged at 5,000 × *g* for 5 min. The pellet of each sample was resuspended in 1.5 mL RNAprotect Bacteria Reagent (QIAGEN, Cat#76506) and incubated and centrifuged according to the manufacturer’s recommendations. The pellet was shock frozen in liquid nitrogen, and samples were stored at −80°C until RNA purification.

### RNA purification

RNA was extracted using an RNeasy Mini Kit (QIAGEN, Cat#74104) with the following modifications. Cell pellets were incubated in 100 mM TRIS-HCL with 800 ng/μL lysostaphin for 25 min at 37°C. Next, 700 μl of RLT buffer containing β-mercaptoethanol was added, and the samples were transferred to lysing matrix B tubes (Fisher Scientific, Cat#MP116911050). After 3 bead beating steps (30 s × 3, with 1 min on ice between each), RNA extraction was followed according to the standard Qiagen RNA extraction protocol. Purified RNA was treated with the Turbo DNA-free Kit (Invitrogen, Cat#AM1907) according to manufacturer’s instructions. RNA concentrations were determined with a Nanodrop spectrophotometer (Thermo Fisher Scientific), and RNA quality control for all RNA-seq samples was performed at the Genomics Core at the University of Colorado Anschutz Medical Campus using an RNA ScreenTape resulting in an RNA integrity number (RIN) between 8.9 and 9.2 for all samples.

### RNA-seq

Depletion of ribosomal RNA and library preparations were performed at the Genomics Core at the University of Colorado Anschutz Medical Campus using the Illumina Ribo-Zero Plus rRNA depletion kit (Illumina, Cat#20037135) and the Zymo-Seq RiboFree total RNA library kit (Zymo Research, Cat#R3003). Sequencing (150 base pairs, paired-end) was performed on an Illumina NovaSEQ 6000. Raw sequencing reads in fastq format were imported into the CLC Genomics Workbench (QIAGEN, version 20.0.4), adapter sequences were trimmed (automatic removal of read-through adapter sequences, removal of low-quality sequence (limit = 0.05)), and trimmed sequences were mapped to the USA300 FPR3757 reference genome (NC_007793) using Qiagen CLC Genomics Workbench default settings (mismatch cost: 2, insertion cost: 3, deletion cost: 3, length fraction: 0.8, similarity fraction: 0.8). Count tables were prefiltered to keep only rows that have a count of at least 10 and were then analyzed for differential gene expression of samples using RStudio (version 2022.07.0, RRID:SCR_000432) and the R package DESeq2 (version 4.2, RRID:SCR_015687) [62] and visualized with R package pheatmap (version 0.2, RRID:SCR_016418). The threshold for differential expressed genes was set at >1 or <−1 Log2 fold change (L2FC) using an adjusted *p*-value (pADJ) of 0.01.

### Murine SSTI model

To prepare bacterial inocula for infection, MRSA USA300 LAC* WT, Δ*camS*, *camS*_Δ69–391_, Δ*camS*::*camS*, *hla*::ΦNƩ, and *hla*::ΦNƩ Δ*camS* were grown overnight in TSB (37°C, shaking at 250 rpm). The following day, all cultures were subcultured to an OD_600_ of 0.05 in fresh TSB and allowed to grow to exponential phase (OD_600_ 0.4 to 0.6) at 37°C and shaking at 250 rpm. Bacterial cells were washed and pelleted in PBS and resuspended in PBS to reach an inoculum of approximately 1 × 10^8^ CFU in 50 μL, which was verified by colony counting after 24 h of incubation at 37°C. Mouse infections were performed as previously described [63]. Briefly, 1 day prior challenge male or female BALB/cJ mice abdomen were shaved, and residual hair was removed with a 30-s application of Nair Hair Remover Lotion (Church & Dwight). Immediately prior to injection, abdomens were sanitized with alcohol wipes, and bacteria were injected intradermally. Lesion size on skin and mouse body weights were measured before infection and every day thereafter for a period of 6 days. Digital images of the skin lesions were taken using a Canon PowerShot ELPH 180 camera and analyzed with FIJI-ImageJ (NIH) software [64]. At day 6 postinfection, the skin lesions were collected with a 12-mm diameter biopsy punch (Fisher Scientific, Cat#NC9253254), weighted, and homogenized in PBS with 1-mm zircona/silica beads (BioSpec Products, Cat#NC19847287) in 3 bead beating steps (1 min × 3, with 1 min on ice between each) with a Mini-Beadbeater-16 (BioSpec). Homogenates were serially diluted in PBS and plated on mannitol salt agar (MSA, BD Cat#211407) supplemented with 5.2 μg/mL cefoxitin to determine CFU counts.

### Dot blots

To detect Hla protein in mouse skin lesions, tissue homogenates were centrifuged twice at 5,000 × *g* for 20 min and normalized to 1 × 10^8^ CFU/gram tissue followed by 2-fold dilutions in PBS. Then, 20 μl of the mouse homogenates from SSTI experiments or 20 μl of a 2-fold dilution of recombinant Hla protein from *S*. *aureus* (0.5 mg/mL in H_2_O, Millipore Sigma, Cat#H9395) were spotted on a 0.45-μm nitrocellulose membrane (BIO-RAD, Cat#1620117) using a Bio-Dot microfiltration apparatus (BIO-RAD, Cat#1706545). The membrane was blocked overnight with Intercept blocking buffer Tris-buffered saline (TBS, LI-COR, Cat#92760001) containing 5% albumin human serum (Calbiochem, Cat#12667) to prevent nonspecific antibody binding. After blocking, the membrane was incubated for 1 ½ hours with a polyclonal rabbit anti-Hla antibody (gifted by Patrick M. Schlievert) diluted 1:5,000 in Intercept buffer + 0.2% Tween 20 + 1% human serum. The membrane was washed 3 times with TSB containing 0.1% Tween 20 (TBST) and incubated with goat anti-rabbit antibodies (IRDye 800CW conjugated, LI-COR Biosciences, Cat#926–32211) diluted 1:15,000 in Intercept buffer TBS with 0.1% Tween 20 and 1% human serum for 1 h at RT. The membrane was washed 4 times with TBST for 5 min each and then imaged on a LiCor Odyssey CLx Imaging System (LI-COR Biosciences) and analyzed with Image Studio Lite (version 5.2, LI-COR).

### In vivo murine sepsis model

Bacterial inoculum for infection was prepared as described for the murine SSTI model with a final inoculum of 1.2 × 10^7^ CFU in 100 μL for each bacterial strain, which was verified by colony counting after 24 h of incubation at 37°C. Female, 7-week-old C57BL/6J mice were then infected via retro-orbital injection of either MRSA USA300 WT or Δ*camS*, and the infection was allowed to proceed for 72 h. Mouse weights were recorded at 24-h intervals, and mice were killed with CO_2_ if their body weight was below 75% or at the completion of the experiment. For bacterial enumeration, organs (kidneys, liver) were harvested, weighted, and homogenized in PBS as described for the murine SSTI model. Homogenates were serially diluted in PBS and plated on MSA supplemented with 5.2 μg/mL cefoxitin to determine CFU counts.

### Generation of dendrogram

The dendrogram of CamS (SAUSA300_1884) orthologs was created based on KEGG SSDB (Sequence Similarity DataBase) database entries [65]. The top 50 orthologs of SAUSA300_1884 were determined with the following settings: Show (Best), Threshold (100), Top50, and the dendrogram was created with the “Create Dendrogram (single)” function.

### Statistical analysis

Statistical analyses were performed using Prism 9 (GraphPad, version 9.4.1). The performed statistical tests are described in the figure legends.

## Supporting information

S1 Fig **(A)** *E*. *faecalis* JH2-2 pAM373::Tn*918* aggregation (lower turbidity) in response to culture filtrates of MRSA USA300 LAC* WT, Δ*camS*::*camS*, and *camS*_Δ69–391_ but not Δ*camS*, both peptide mutants (*camS*_F13A, I14V_ and *camS*_L15V_) or medium control. AU = arbitrary unit (*n* = 6). **(Bi)** Schematic of *camS* gene locus (SAUSA300_1884), displaying the putative TSS. RNA-seq data from MRSA USA300 was used to identify the *camS* TSS and putative promoter region in MRSA USA300. **(ii)** Quantitative determination of the RFU in P_*camS*_-DsRed expressing WT over the course of 48 h (*n* = 4). **(Ci)** 24 h growth curves (*n* = 4) and **(ii)** growth yield (CFU/mL) of WT, Δ*camS*, Δ*camS*::*camS*, *camS*_Δ69–391_, *camS*_F13A,I14V_, and *camS*_L15V_ (*n* = 3, individual dots). **(D)** Dendrogram of CamS (SAUSA300_1884) orthologs in staphylococcal species. The dendrogram was generated based on KEGG SSDB (Sequence Similarity DataBase) entries. **(E)** Quantitative determination of the RFU in **(i)** P_*camS*_-DsRed-expressing MRSA USA300 WT and MRSA USA400 WT strains after 24 h (*n* = 4). Quantitative determination of the RFU in P_*hla*_-DsRed-expressing **(ii)** USA300 WT and USA300 Δ*camS* and **(iii)** USA400 WT and USA400 Δ*camS* after 24 h (*n* = 4). Results represent the pooled data from independent experiments (individual dots), and all data are shown as mean ± SD. Significant differences were determined by one-way ANOVA with Bonferroni’s (**A**) and Dunnett’s (**Cii**) multiple comparisons test or by an unpaired *t* test (**Eii, Eiii **). ****P < 0.0001, ns = not significant. The data underlying panels A, B, C, and E can be found in S1 Data. CFU, colony-forming unit; MRSA, methicillin-resistant *S*. *aureus*; RFU, relative fluorescence unit; RNA-seq, RNA-sequencing; TSS, transcriptional start site; WT, wild type.
(TIF)

S2 Fig **(Ai)** Representative images of dermonecrotic lesion size in mice (BALB/cJ) after 6 days postinfection with MRSA USA300 LAC* WT, *hla*::ΦNƩ, and *hla*::ΦNƩ Δ*camS*. **(ii)** Dermonecrotic lesion size and weight change of mice following infection with WT, *hla*::ΦNƩ, and *hla*::ΦNƩ Δ*camS* over the course of 6 days postinfection. Data are shown as mean ± SD (13 mice per group). **(iii)** Bacterial burden, measured as CFU/gram (CFU/g) in homogenized lesions at day 6 postinfection for the indicated groups. Data were pooled from 4 independent experiments and presented as median with interquartile range (individual dots *=* mice, 13 per group). **(iv)** The weight change of mice infected with WT or mutant strains at 6 days postinfection. Data were pooled from 6 independent experiments and presented as median with interquartile range (individual dots = mice, 20 for WT, 22 for Δ*camS*, 21 for Δ*camS*::*camS*, 20 for *camS*_Δ69–391_). Significant differences were determined by a Kruskal–Wallis test followed by a post hoc Dunn’s multiple comparison test. ns = not significant. **(B)** Dot immunoblot of homogenized tissues from dermonecrotic lesions (day 6) from mice infected with WT, Δ*camS*, Δ*camS*::*camS*, and *camS*_Δ69–391_ strains. Two-fold dilutions of the homogenates or recombinant Hla protein from *S*. *aureus* (9.4–300 ng) were spotted on a nitrocellulose membrane and probed using Hla antibodies. Shown are 3 sets from independent experiments. Set 2, marked with a rectangle, was used as a representative image in Fig 2C. **(C)** Bacterial CFU/gram (CFU/g) was determined in **(i)** the kidney and **(ii)** liver of mice (C57BL/6J) infected with the MRSA USA300 LAC* WT or the Δ*camS* mutant in a murine sepsis model. Mouse organs were harvested and homogenized at the day of death. Data were pooled from 2 independent experiments and presented as median with interquartile range (individual dots = mice, 10 for WT, 12 for Δ*camS*). Significant differences in (**Aiii**) and (**C**) were determined by a Mann–Whitney *U* test, ns = not significant. The data underlying panels A and C can be found in S1 Data. CFU, colony-forming unit; MRSA, methicillin-resistant *S*. *aureus*; WT, wild type.
(TIF)

S1 Data Numerical data underlying Figs 1, 2, S1 and S2. (XLSX)

S1 Table Excel file containing RNA-seq results (Δ*camS* versus WT, *camS*Δ69–391 versus WT, Δ*camS* versus Δ*camS*::*camS*). (XLSX)

S2 Table RBC hemolysis (EC_50_). (DOCX)

S3 Table Bacterial strains and plasmids used in this study. (DOCX)

S4 Table Primers used in this study. (DOCX)

## Abbreviations

ADAM10: A Disintegrin and Metalloproteinase 10
CDM: chemically defined medium
CFU: colony-forming unit
FBS: fetal bovine serum
HGT: horizontal gene transfer
hPMN: human polymorphonuclear leukocytes
LDH: lactate dehydrogenase
Lpp: lipoproteins
MRSA: methicillin-resistant *S*. *aureus*
PBS: phosphate-buffered saline
RBC: red blood cells
RFU: relative fluorescence unit
RIN: RNA integrity number
RNA-seq: RNA-sequencing
RT: room temperature
Sae: *S*. *aureus* exoprotein
SSTI: skin and soft tissue infection
TBS: Tris-buffered saline
TCS: two-component system
TLR2: Toll-like receptor 2
TSA: tryptic soy agar
TSB: tryptic soy broth
WT: wild type

## References

[1] Williams RE. Healthy carriage of *Staphylococcus aureus*: its prevalence and importance. Bacteriol Rev. 1963;27(1):56–71. 14000926 10.1128/br.27.1.56-71.1963 PMC441169

[2] Wertheim HF, Melles DC, Vos MC, van Leeuwen W, van Belkum A, Verbrugh HA, et al. The role of nasal carriage in *Staphylococcus aureus* infections. Lancet Infect Dis. 2005;5(12):751–762. 16310147 10.1016/S1473-3099(05)70295-4

[3] Lowy FD. *Staphylococcus aureus* infections. N Engl J Med. 1998;339(8):520–532. 9709046 10.1056/NEJM199808203390806

[4] Tong SY, Davis JS, Eichenberger E, Holland TL, Fowler VG Jr. *Staphylococcus aureus* infections: epidemiology, pathophysiology, clinical manifestations, and management. Clin Microbiol Rev. 2015;28(3):603–661. 26016486 10.1128/CMR.00134-14 PMC4451395

[5] Bleul L, Francois P, Wolz C. Two-Component Systems of *S*. *aureus*: Signaling and Sensing Mechanisms. Genes (Basel). 2021;13(1):34. 35052374 10.3390/genes13010034 PMC8774646

[6] Diep BA, Phung Q, Date S, Arnott D, Bakalarski C, Xu M, et al. Identifying potential therapeutic targets of methicillin-resistant *Staphylococcus aureus* through in vivo proteomic analysis. J Infect Dis. 2014;209(10):1533–1541. 24280367 10.1093/infdis/jit662 PMC3997574

[7] Braun V, Hantke K. Lipoproteins: Structure, Function. Biosynthesis. Subcell Biochem. 2019;92:39–77. 31214984 10.1007/978-3-030-18768-2_3

[8] Nguyen MT, Götz F. Lipoproteins of Gram-Positive Bacteria: Key Players in the Immune Response and Virulence. Microbiol Mol Biol Rev. 2016;80(3):891–903. doi: 10.1128/MMBR.00028-16 27512100 PMC4981669

[9] Nguyen MT, Matsuo M, Niemann S, Herrmann M, Götz F. Lipoproteins in Gram-Positive Bacteria: Abundance, Function, Fitness. Front Microbiol. 2020;11:582582. doi: 10.3389/fmicb.2020.582582 33042100 PMC7530257

[10] Shahmirzadi SV, Nguyen MT, Götz F. Evaluation of *Staphylococcus aureus* Lipoproteins: Role in Nutritional Acquisition and Pathogenicity. Front Microbiol. 2016;7:1404. 27679612 10.3389/fmicb.2016.01404 PMC5020093

[11] Brightbill HD, Libraty DH, Krutzik SR, Yang RB, Belisle JT, Bleharski JR, et al. Host defense mechanisms triggered by microbial lipoproteins through toll-like receptors. Science. 1999;285(5428):732–736. doi: 10.1126/science.285.5428.732 10426995

[12] Aliprantis AO, Yang RB, Mark MR, Suggett S, Devaux B, Radolf JD, et al. Cell activation and apoptosis by bacterial lipoproteins through toll-like receptor-2. Science. 1999;285(5428):736–739. doi: 10.1126/science.285.5428.736 10426996

[13] Hashimoto M, Tawaratsumida K, Kariya H, Aoyama K, Tamura T, Suda Y. Lipoprotein is a predominant Toll-like receptor 2 ligand in *Staphylococcus aureus* cell wall components. Int Immunol. 2006;18(2):355–362. 16373361 10.1093/intimm/dxh374

[14] Nguyen MT, Uebele J, Kumari N, Nakayama H, Peter L, Ticha O, et al. Lipid moieties on lipoproteins of commensal and non-commensal staphylococci induce differential immune responses. Nat Commun. 2017;8(1):2246. doi: 10.1038/s41467-017-02234-4 29269769 PMC5740139

[15] Dunny GM, Berntsson RP. Enterococcal Sex Pheromones: Evolutionary Pathways to Complex, Two-Signal Systems. J Bacteriol. 2016;198(11):1556–1562. doi: 10.1128/JB.00128-16 27021562 PMC4959283

[16] Schilcher K, Caesar LK, Cech NB, Horswill AR. Processing, Export, and Identification of Novel Linear Peptides from *Staphylococcus aureus*. mBio. 2020;11(2):e00112–e00120. 32291297 10.1128/mBio.00112-20 PMC7157817

[17] Xayarath B, Alonzo F 3rd, Freitag NE. Identification of a peptide-pheromone that enhances *Listeria monocytogenes* escape from host cell vacuoles. PLoS Pathog. 2015;11(3):e1004707. 25822753 10.1371/journal.ppat.1004707 PMC4379056

[18] Remy L, Carrière M, Derré-Bobillot A, Martini C, Sanguinetti M, Borezée-Durant E. The *Staphylococcus aureus* Opp1 ABC transporter imports nickel and cobalt in zinc-depleted conditions and contributes to virulence. Mol Microbiol. 2013;87(4):730–743. 23279021 10.1111/mmi.12126

[19] DeLeo FR, Otto M, Kreiswirth BN, Chambers HF. Community-associated meticillin-resistant *Staphylococcus aureus*. Lancet. 2010;375(9725):1557–1568. 20206987 10.1016/S0140-6736(09)61999-1 PMC3511788

[20] Clewell DB, An FY, White BA, Gawron-Burke C. *Streptococcus faecalis* sex pheromone (cAM373) also produced by *Staphylococcus aureus* and identification of a conjugative transposon (Tn*918*). J Bacteriol. 1985;162(3):1212–1220. 2987186 10.1128/jb.162.3.1212-1220.1985 PMC215906

[21] Prados J, Linder P, Redder P. TSS-EMOTE, a refined protocol for a more complete and less biased global mapping of transcription start sites in bacterial pathogens. BMC Genomics. 2016;17(1):849. doi: 10.1186/s12864-016-3211-3 27806702 PMC5094136

[22] Giraudo AT, Calzolari A, Cataldi AA, Bogni C, Nagel R. The *sae* locus of *Staphylococcus aureus* encodes a two-component regulatory system. FEMS Microbiol Lett. 1999;177(1):15–22. 10436918 10.1111/j.1574-6968.1999.tb13707.x

[23] Jeong DW, Cho H, Jones MB, Shatzkes K, Sun F, Ji Q, et al. The auxiliary protein complex SaePQ activates the phosphatase activity of sensor kinase SaeS in the SaeRS two-component system of *Staphylococcus aureus*. Mol Microbiol. 2012;86(2):331–348. 22882143 10.1111/j.1365-2958.2012.08198.x PMC3468659

[24] Nygaard TK, Pallister KB, Ruzevich P, Griffith S, Vuong C, Voyich JM. SaeR binds a consensus sequence within virulence gene promoters to advance USA300 pathogenesis. J Infect Dis. 2010;201(2):241–254. doi: 10.1086/649570 20001858 PMC2798008

[25] Sun F, Li C, Jeong D, Sohn C, He C, Bae T. In the *Staphylococcus aureus* two-component system *sae*, the response regulator SaeR binds to a direct repeat sequence and DNA binding requires phosphorylation by the sensor kinase SaeS. J Bacteriol. 2010;192(8):2111–2127. 20172998 10.1128/JB.01524-09 PMC2849438

[26] Cooney J, Kienle Z, Foster TJ, O’Toole PW. The gamma-hemolysin locus of *Staphylococcus aureus* comprises three linked genes, two of which are identical to the genes for the F and S components of leukocidin. Infect Immun. 1993;61(2):768–771. 8423103 10.1128/iai.61.2.768-771.1993 PMC302792

[27] Montgomery CP, Boyle-Vavra S, Adem PV, Lee JC, Husain AN, Clasen J, et al. Comparison of virulence in community-associated methicillin-resistant *Staphylococcus aureus* pulsotypes USA300 and USA400 in a rat model of pneumonia. J Infect Dis. 2008;198(4):561–570. 18598194 10.1086/590157

[28] Kennedy AD, Bubeck Wardenburg J, Gardner DJ, Long D, Whitney AR, Braughton KR, et al. Targeting of alpha-hemolysin by active or passive immunization decreases severity of USA300 skin infection in a mouse model. J Infect Dis. 2010;202(7):1050–1058. doi: 10.1086/656043 20726702 PMC2945289

[29] Sampedro GR, DeDent AC, Becker RE, Berube BJ, Gebhardt MJ, Cao H, et al. Targeting *Staphylococcus aureus* alpha-toxin as a novel approach to reduce severity of recurrent skin and soft-tissue infections. J Infect Dis. 2014;210(7):1012–1018. 24740631 10.1093/infdis/jiu223 PMC4207862

[30] DuMont AL, Yoong P, Day CJ, Alonzo F 3rd, McDonald WH, Jennings MP, et al. *Staphylococcus aureus* LukAB cytotoxin kills human neutrophils by targeting the CD11b subunit of the integrin Mac-1. Proc Natl Acad Sci U S A. 2013;110(26):10794–10799. 23754403 10.1073/pnas.1305121110 PMC3696772

[31] Spaan AN, Vrieling M, Wallet P, Badiou C, Reyes-Robles T, Ohneck EA, et al. The staphylococcal toxins gamma-haemolysin AB and CB differentially target phagocytes by employing specific chemokine receptors. Nat Commun. 2014;5:5438. 25384670 10.1038/ncomms6438 PMC4228697

[32] Rigby KM, DeLeo FR. Neutrophils in innate host defense against *Staphylococcus aureus* infections. Semin Immunopathol. 2012;34(2):237–259. 22080185 10.1007/s00281-011-0295-3 PMC3271231

[33] Kwiecinski JM, Horswill AR. *Staphylococcus aureus* bloodstream infections: pathogenesis and regulatory mechanisms. Curr Opin Microbiol. 2020;53:51–60. 32172183 10.1016/j.mib.2020.02.005 PMC7244392

[34] Cheung GYC, Bae JS, Liu R, Hunt RL, Zheng Y, Otto M. Bacterial virulence plays a crucial role in MRSA sepsis. PLoS Pathog. 2021;17(2):e1009369. doi: 10.1371/journal.ppat.1009369 33630954 PMC7942999

[35] von Eiff C, Friedrich AW, Peters G, Becker K. Prevalence of genes encoding for members of the staphylococcal leukotoxin family among clinical isolates of *Staphylococcus aureus*. Diagn Microbiol Infect Dis. 2004;49(3):157–162. 15246504 10.1016/j.diagmicrobio.2004.03.009

[36] Prevost G, Couppie P, Prevost P, Gayet S, Petiau P, Cribier B, et al. Epidemiological data on *Staphylococcus aureus* strains producing synergohymenotropic toxins. J Med Microbiol. 1995;42(4):237–245. 7707330 10.1099/00222615-42-4-237

[37] McCarthy AJ, Lindsay JA. *Staphylococcus aureus* innate immune evasion is lineage-specific: a bioinfomatics study. Infect Genet Evol. 2013;19:7–14. 23792184 10.1016/j.meegid.2013.06.012

[38] Alonzo F 3rd, Torres VJ. The bicomponent pore-forming leucocidins of *Staphylococcus aureus*. Microbiol Mol Biol Rev. 2014;78(2):199–230. 24847020 10.1128/MMBR.00055-13 PMC4054254

[39] Buelow DR, Raivio TL. Three (and more) component regulatory systems—auxiliary regulators of bacterial histidine kinases. Mol Microbiol. 2010;75(3):547–566. doi: 10.1111/j.1365-2958.2009.06982.x 19943903

[40] Göpel Y, Görke B. Interaction of lipoprotein QseG with sensor kinase QseE in the periplasm controls the phosphorylation state of the two-component system QseE/QseF in *Escherichia coli*. PLoS Genet. 2018;14(7):e1007547. 30040820 10.1371/journal.pgen.1007547 PMC6075780

[41] Wilke GA, Bubeck WJ. Role of a disintegrin and metalloprotease 10 in *Staphylococcus aureus* alpha-hemolysin-mediated cellular injury. Proc Natl Acad Sci U S A. 2010;107(30):13473–13478. 20624979 10.1073/pnas.1001815107 PMC2922128

[42] Thammavongsa V, Kim HK, Missiakas D, Schneewind O. Staphylococcal manipulation of host immune responses. Nat Rev Microbiol. 2015;13(9):529–543. doi: 10.1038/nrmicro3521 26272408 PMC4625792

[43] Zheng X, Marsman G, Lacey KA, Chapman JR, Goosmann C, Ueberheide BM, et al. The cell envelope of *Staphylococcus aureus* selectively controls the sorting of virulence factors. Nat Commun. 2021;12(1):6193. 34702812 10.1038/s41467-021-26517-z PMC8548510

[44] Zheng X, Ma SX, St John A, Torres VJ. The Major Autolysin Atl Regulates the Virulence of *Staphylococcus aureus* by Controlling the Sorting of LukAB. Infect Immun. 2022;90(4):e0005622. 35258336 10.1128/iai.00056-22 PMC9022505

[45] Talan DA, Krishnadasan A, Gorwitz RJ, Fosheim GE, Limbago B, Albrecht V, et al. Comparison of *Staphylococcus aureus* from skin and soft-tissue infections in US emergency department patients, 2004 and 2008. Clin Infect Dis. 2011;53(2):144–149. 21690621 10.1093/cid/cir308

[46] Berube BJ, Bubeck WJ. *Staphylococcus aureus* alpha-toxin: nearly a century of intrigue. Toxins (Basel). 2013;5(6):1140–1166. 23888516 10.3390/toxins5061140 PMC3717774

[47] Popov LM, Marceau CD, Starkl PM, Lumb JH, Shah J, Guerrera D, et al. The adherens junctions control susceptibility to *Staphylococcus aureus* alpha-toxin. Proc Natl Acad Sci U S A. 2015;112(46):14337–14342. 26489655 10.1073/pnas.1510265112 PMC4655540

[48] Malachowa N, Whitney AR, Kobayashi SD, Sturdevant DE, Kennedy AD, Braughton KR, et al. Global changes in *Staphylococcus aureus* gene expression in human blood. PLoS ONE. 2011;6(4):e18617. 21525981 10.1371/journal.pone.0018617 PMC3078114

[49] Nilsson IM, Hartford O, Foster T, Tarkowski A. Alpha-toxin and gamma-toxin jointly promote *Staphylococcus aureus* virulence in murine septic arthritis. Infect Immun. 1999;67(3):1045–1049. 10024541 10.1128/iai.67.3.1045-1049.1999 PMC96427

[50] Haslett C, Guthrie LA, Kopaniak MM, Johnston RB Jr, Henson PM. Modulation of multiple neutrophil functions by preparative methods or trace concentrations of bacterial lipopolysaccharide. Am J Pathol. 1985;119(1):101–110. 2984939 PMC1888083

[51] Novick RP. Genetic systems in staphylococci. Methods Enzymol. 1991;204:587–636. doi: 10.1016/0076-6879(91)04029-n 1658572

[52] Fey PD, Endres JL, Yajjala VK, Widhelm TJ, Boissy RJ, Bose JL, et al. A genetic resource for rapid and comprehensive phenotype screening of nonessential *Staphylococcus aureus* genes. mBio. 2013;4(1):e00537–e00512. 23404398 10.1128/mBio.00537-12 PMC3573662

[53] Löfblom J, Kronqvist N, Uhlén M, Ståhl S, Wernérus H. Optimization of electroporation-mediated transformation: *Staphylococcus carnosus* as model organism. J Appl Microbiol. 2007;102(3):736–747. 17309623 10.1111/j.1365-2672.2006.03127.x

[54] Monk IR, Shah IM, Xu M, Tan MW, Foster TJ. Transforming the untransformable: application of direct transformation to manipulate genetically *Staphylococcus aureus* and *Staphylococcus epidermidis*. mBio. 2012;3(2):e00277–e00211. 22434850 10.1128/mBio.00277-11 PMC3312211

[55] Crosby HA, Schlievert PM, Merriman JA, King JM, Salgado-Pabón W, Horswill AR. The *Staphylococcus aureus* Global Regulator MgrA Modulates Clumping and Virulence by Controlling Surface Protein Expression. PLoS Pathog. 2016;12(5):e1005604. 27144398 10.1371/journal.ppat.1005604 PMC4856396

[56] Liu H, Naismith JH. An efficient one-step site-directed deletion, insertion, single and multiple-site plasmid mutagenesis protocol. BMC Biotechnol. 2008;8:91. doi: 10.1186/1472-6750-8-91 19055817 PMC2629768

[57] Parlet CP, Kavanaugh JS, Crosby HA, Raja HA, El-Elimat T, Todd DA, et al. Apicidin Attenuates MRSA Virulence through Quorum-Sensing Inhibition and Enhanced Host Defense. Cell Rep. 2019;27(1):187–98.e6. doi: 10.1016/j.celrep.2019.03.018 30943400 PMC7224364

[58] Ibberson CB, Parlet CP, Kwiecinski J, Crosby HA, Meyerholz DK, Horswill AR. Hyaluronan Modulation Impacts *Staphylococcus aureus* Biofilm Infection. Infect Immun. 2016;84(6):1917–1929. 27068096 10.1128/IAI.01418-15 PMC4907140

[59] Dunny GM, Craig RA, Carron RL, Clewell DB. Plasmid transfer in *Streptococcus faecalis*: production of multiple sex pheromones by recipients. Plasmid. 1979;2(3):454–465. 113798 10.1016/0147-619x(79)90029-5

[60] Ibberson CB, Jones CL, Singh S, Wise MC, Hart ME, Zurawski DV, et al. *Staphylococcus aureus* hyaluronidase is a CodY-regulated virulence factor. Infect Immun. 2014;82(10):4253–4264. 25069977 10.1128/IAI.01710-14 PMC4187871

[61] Quave CL, Lyles JT, Kavanaugh JS, Nelson K, Parlet CP, Crosby HA, et al. *Castanea sativa* (European Chestnut) Leaf Extracts Rich in Ursene and Oleanene Derivatives Block *Staphylococcus aureus* Virulence and Pathogenesis without Detectable Resistance. PLoS ONE. 2015;10(8):e0136486. 26295163 10.1371/journal.pone.0136486 PMC4546677

[62] Love MI, Huber W, Anders S. Moderated estimation of fold change and dispersion for RNA-seq data with DESeq2. Genome Biol. 2014;15(12):550. doi: 10.1186/s13059-014-0550-8 25516281 PMC4302049

[63] Severn MM, Williams MR, Shahbandi A, Bunch ZL, Lyon LM, Nguyen A, et al. The Ubiquitous Human Skin Commensal *Staphylococcus hominis* Protects against Opportunistic Pathogens. mBio. 2022;13(3):e0093022. 35608301 10.1128/mbio.00930-22 PMC9239047

[64] Schindelin J, Arganda-Carreras I, Frise E, Kaynig V, Longair M, Pietzsch T, et al. Fiji: an open-source platform for biological-image analysis. Nat Methods. 2012;9(7):676–682. doi: 10.1038/nmeth.2019 22743772 PMC3855844

[65] Kanehisa M, Goto S. KEGG: kyoto encyclopedia of genes and genomes. Nucleic Acids Res. 2000;28(1):27–30. doi: 10.1093/nar/28.1.27 10592173 PMC102409

