# Supplementary figures and images for: The Staphylococcus aureus CamS lipoprotein is a repressor of toxin production that shapes host-pathogen interaction
Source: PLoS Biol. 2024 Jan 5;22(1):e3002451. doi: 10.1371/journal.pbio.3002451 (PMC10769083; doi:10.1371/journal.pbio.3002451)

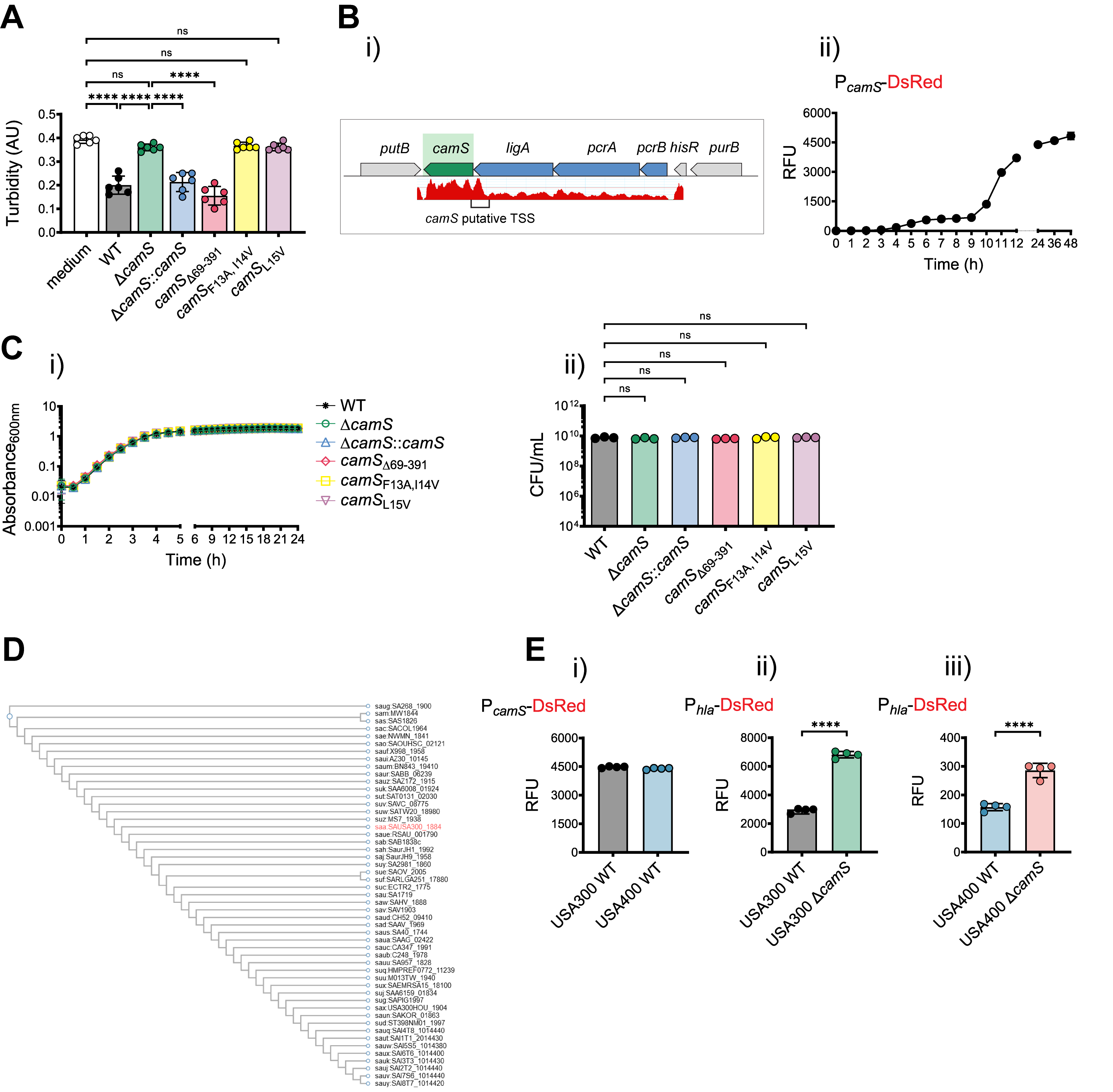

Supplement: S1 Fig — (A) E. faecalis JH2-2 pAM373::Tn918 aggregation (lower turbidity) in response to culture filtrates of MRSA USA300 LAC* WT, ΔcamS::camS, and camSΔ69–391 but not ΔcamS, both peptide mutants (camSF13A, I14V and camSL15V) or medium control. AU = arbitrary unit (n = 6). (Bi) Schematic of camS gene locus (SAUSA300_1884), displaying the putative TSS. RNA-seq data from MRSA USA300 was used to identify the camS TSS and putative promoter region in MRSA USA300. (ii) Quantitative determination of the RFU in PcamS-DsRed expressing WT over the course of 48 h (n = 4). (Ci) 24 h growth curves (n = 4) and (ii) growth yield (CFU/mL) of WT, ΔcamS, ΔcamS::camS, camSΔ69–391, camSF13A,I14V, and camSL15V (n = 3, individual dots). (D) Dendrogram of CamS (SAUSA300_1884) orthologs in staphylococcal species. The dendrogram was generated based on KEGG SSDB (Sequence Similarity DataBase) entries. (E) Quantitative determination of the RFU in (i) PcamS-DsRed-expressing MRSA USA300 WT and MRSA USA400 WT strains after 24 h (n = 4). Quantitative determination of the RFU in Phla-DsRed-expressing (ii) USA300 WT and USA300 ΔcamS and (iii) USA400 WT and USA400 ΔcamS after 24 h (n = 4). Results represent the pooled data from independent experiments (individual dots), and all data are shown as mean ± SD. Significant differences were determined by one-way ANOVA with Bonferroni’s (A) and Dunnett’s (Cii) multiple comparisons test or by an unpaired t test (Eii, Eiii ). ****P < 0.0001, ns = not significant. The data underlying panels A, B, C, and E can be found in S1 Data. CFU, colony-forming unit; MRSA, methicillin-resistant S. aureus; RFU, relative fluorescence unit; RNA-seq, RNA-sequencing; TSS, transcriptional start site; WT, wild type. (TIF) [file pbio.3002451.s001.tif]

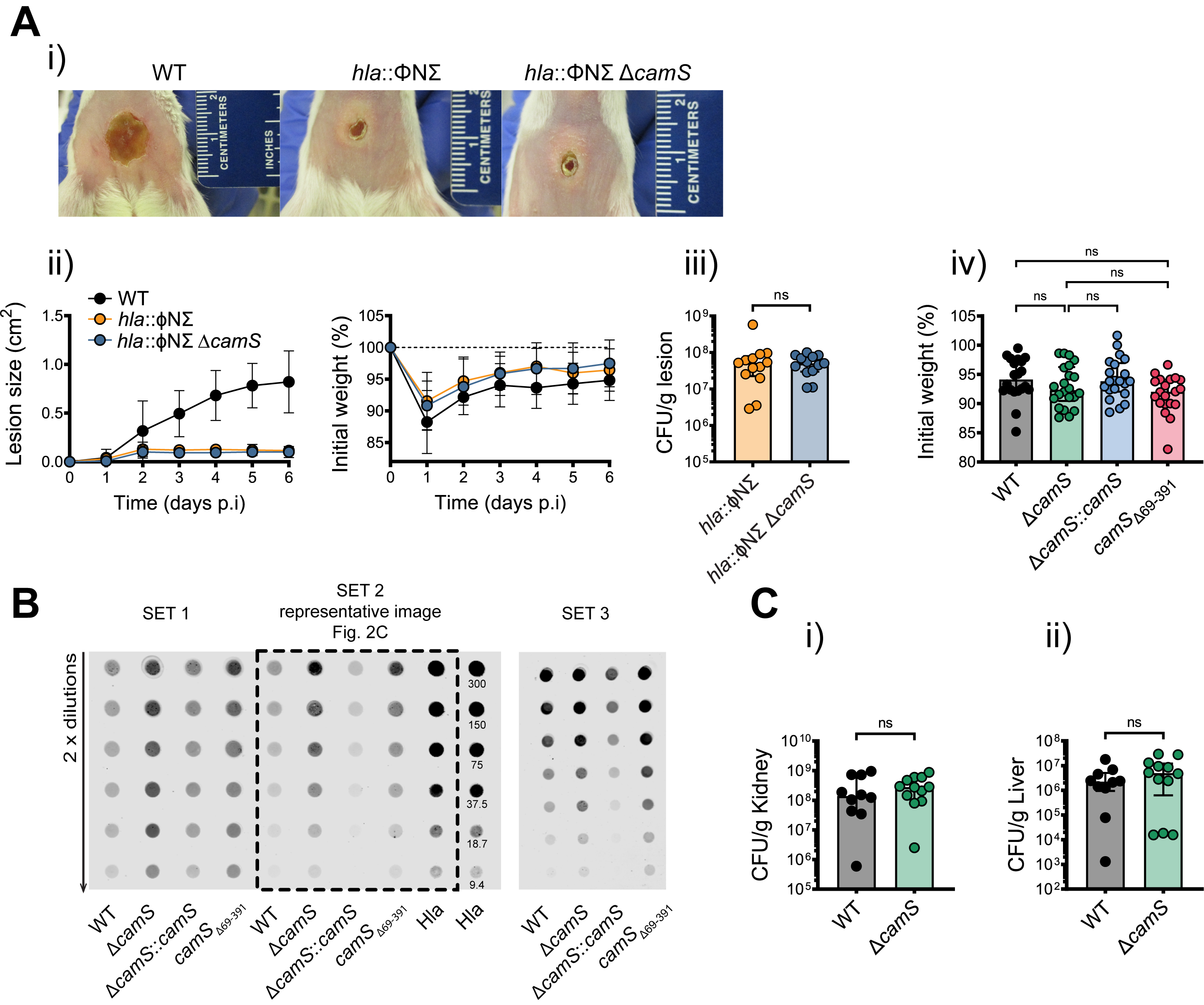

Supplement: S2 Fig — (Ai) Representative images of dermonecrotic lesion size in mice (BALB/cJ) after 6 days postinfection with MRSA USA300 LAC* WT, hla::ΦNƩ, and hla::ΦNƩ ΔcamS. (ii) Dermonecrotic lesion size and weight change of mice following infection with WT, hla::ΦNƩ, and hla::ΦNƩ ΔcamS over the course of 6 days postinfection. Data are shown as mean ± SD (13 mice per group). (iii) Bacterial burden, measured as CFU/gram (CFU/g) in homogenized lesions at day 6 postinfection for the indicated groups. Data were pooled from 4 independent experiments and presented as median with interquartile range (individual dots = mice, 13 per group). (iv) The weight change of mice infected with WT or mutant strains at 6 days postinfection. Data were pooled from 6 independent experiments and presented as median with interquartile range (individual dots = mice, 20 for WT, 22 for ΔcamS, 21 for ΔcamS::camS, 20 for camSΔ69–391). Significant differences were determined by a Kruskal–Wallis test followed by a post hoc Dunn’s multiple comparison test. ns = not significant. (B) Dot immunoblot of homogenized tissues from dermonecrotic lesions (day 6) from mice infected with WT, ΔcamS, ΔcamS::camS, and camSΔ69–391 strains. Two-fold dilutions of the homogenates or recombinant Hla protein from S. aureus (9.4–300 ng) were spotted on a nitrocellulose membrane and probed using Hla antibodies. Shown are 3 sets from independent experiments. Set 2, marked with a rectangle, was used as a representative image in Fig 2C. (C) Bacterial CFU/gram (CFU/g) was determined in (i) the kidney and (ii) liver of mice (C57BL/6J) infected with the MRSA USA300 LAC* WT or the ΔcamS mutant in a murine sepsis model. Mouse organs were harvested and homogenized at the day of death. Data were pooled from 2 independent experiments and presented as median with interquartile range (individual dots = mice, 10 for WT, 12 for ΔcamS). Significant differences in (Aiii) and (C) were determined by a Mann–Whitney U test, ns = not significant. The data u [file pbio.3002451.s002.tif]
