# Supplementary material for: The Staphylococcus aureus CamS lipoprotein is a repressor of toxin production that shapes host-pathogen interaction
Source: PLoS Biol. 2024 Jan 5;22(1):e3002451. doi: 10.1371/journal.pbio.3002451 (PMC10769083; doi:10.1371/journal.pbio.3002451)
Supplement: S2 Table — (DOCX) [file pbio.3002451.s005.docx]

**S2 Table: RBC hemolysis (EC_50_)**

EC_50_ values - Percentage of culture filtrate needed to lyse 50% of erythrocytes

|  | **Biological Replicates** | | | | |
| --- | --- | --- | --- | --- | --- |
| **Bacterial Strain** | **1** | **2** | **3** | **4** | **5** |
| LAC* WT | 1.11 | 1.20 | 1.51 | 1.44 | 1.42 |
| LAC* Δ*camS* | 0.87 | 0.80 | 0.84 | 0.77 | 0.84 |
| LAC* Δ*camS*::*camS* | 0.94 | 1.06 | 1.41 | 1.21 | 1.34 |
| LAC* *camS*_Δ69-391_ | 0.72 | 0.81 | 0.91 | 0.81 | 0.89 |
| LAC* *camS*_F13A,I14V_ | 1.05 | 0.99 | 1.41 | 1.39 | 1.34 |
| LAC* *camS*_L15V_ | 1.00 | 1.27 | 1.38 | 1.30 | 1.36 |
| LAC* *hla*::ΦNƩ | 15.58 | 17.58 | 20.30 | 27.68 | 27.76 |
| LAC* *hla*::ΦNƩ Δ*camS* | 9.55 | 10.21 | 22.07 | 12.33 | 10.38 |
