# Supplementary material for: The Staphylococcus aureus CamS lipoprotein is a repressor of toxin production that shapes host-pathogen interaction
Source: PLoS Biol. 2024 Jan 5;22(1):e3002451. doi: 10.1371/journal.pbio.3002451 (PMC10769083; doi:10.1371/journal.pbio.3002451)
Supplement: S3 Table — (DOCX) [file pbio.3002451.s006.docx]

**S3 Table: Bacterial strains and plasmids used in this study**

| STRAINS/ PLASMIDS | SOURCE | IDENTIFIER |
| --- | --- | --- |
| **Bacterial strains** | | |
| *E. coli* DC10B | [1] | N/A |
| *E. coli* Top10 | Invitrogen | N/A |
| *S. aureus* USA300 CA-MRSA LAC, Erm^S^ (= LAC*) | [2] | AH1263 |
| *S. aureus* LAC* Δ*camS* | [3] | AH5583 |
| *S. aureus* USA400 CA-MRSA MW2 | [4] | AH843 |
| *S. aureus* MW2 Δ*camS* | This Paper | AH6094 |
| *S. aureus* LAC* Δ*camS*::*camS* | This Paper | AH6091 |
| *S. aureus* LAC* *camS*_Δ69-391_ | This Paper | AH6088 |
| *S. aureus* LAC* *camS*_F13A,I14V_ | This Paper | AH6090 |
| *S. aureus* LAC* *camS*_L15V_ | This Paper | AH6089 |
| *S. aureus* LAC* Δ*saePQRS* | [5] | AH2216 |
| *S. aureus* LAC* Δ*saePQRS* Δ*camS* | This Paper | AH6092 |
| *S. aureus* LAC* *hla*::ΦNƩ | This Paper | AH5832 |
| *S. aureus* LAC* *hla*::ΦNƩ Δ*camS* | This Paper | AH6093 |
| *S. aureus* LAC* *lukA*::ΦNƩ | This Paper | AH6064 |
| *S. aureus* LAC* *lukA*::ΦNƩ Δ*camS* | This Paper | AH6065 |
| *S. aureus* LAC* *hlgA*::ΦNƩ | This Paper | AH6066 |
| *S. aureus* LAC* *hlgA*::ΦNƩ Δ*camS* | This Paper | AH6067 |
| *S. aureus* LAC* *hlgB*::ΦNƩ | This Paper | AH6068 |
| *S. aureus* LAC* *hlgB*::ΦNƩ Δ*camS* | This Paper | AH6069 |
| *S. aureus* LAC* *hlgC*::ΦNƩ | This Paper | AH6070 |
| *S. aureus* LAC* *hlgC*::ΦNƩ Δ*camS* | This Paper | AH6071 |
| **Plasmids** | | |
| pAM373::Tn*918*; responds to *staph*-cAM373, Tet^R^ | [6] | N/A |
| pJB38; mutation generation vector, Amp^R^/ Cm^R^ | [7] | N/A |
| pKAS57; pJB38-*camS*(USA400) deletion vector, Amp^R^/ Cm^R^ | This Paper | N/A |
| pKAS07; pJB38-*camS*(USA300) deletion vector, Amp^R^/ Cm^R^ | [3] | N/A |
| pKAS61; pJB38-*camS* complementation vector, Amp^R^/ Cm^R^ | This Paper | N/A |
| pKAS63; pJB38-*camS*_Δ69-391_ deletion vector,  Amp^R^/ Cm^R^ | This Paper | N/A |
| pKAS38; pJB38-*camS* expression vector,  Amp^R^/ Cm^R^ | This Paper | N/A |
| pKAS39; pJB38-*camS*_F13A,I14V_ AA exchange vector, Amp^R^/ Cm^R^ | This Paper | N/A |
| pKAS53; pJB38-*camS*_L15V_ AA exchange vector, Amp^R^/ Cm^R^ | This Paper | N/A |
| pCM11; sGFP expression vector, Amp^R^/ Erm^R^ | [8] | N/A |
| pCM28; *S. aureus* – *E. coli* shuttle vector,  Amp^R^/ Cm^R^ | [9] | N/A |
| pCM36; pCM28-*hla* promoter (USA300) sGFP fusion vector, Amp^R^/ Cm^R^ | This Paper | N/A |
| pHC48; DsRed expression vector, Amp^R^/ Cm^R^ | [10] | N/A |
| pKAS108; pHC48-*hla* promoter (USA300) DsRed fusion vector, Amp^R^/ Cm^R^ | This Paper | N/A |
| pKAS109; pHC48-*hla* promoter (USA400) DsRed fusion vector, Amp^R^/ Cm^R^ | This Paper | N/A |
| pKAS44; pHC48-*camS* promoter DsRed fusion vector, Amp^R^/ Cm^R^ | This Paper | N/A |
| pKAS92; pHC48-*lukA* promoter DsRed fusion vector, Amp^R^/ Cm^R^ | This Paper | N/A |
| pKAS103; pHC48-*hlgA* promoter DsRed fusion vector, Amp^R^/ Cm^R^ | This Paper | N/A |
| pKAS105; pHC48-*hlgCB* promoter DsRed fusion vector, Amp^R^/ Cm^R^ | This Paper | N/A |
| pKAS43; pHC48 without promoter, Amp^R^/ Cm^R^ | This Paper | N/A |

References:

1. Monk, I.R., et al., *Transforming the untransformable: application of direct transformation to manipulate genetically Staphylococcus aureus and Staphylococcus epidermidis.* mBio, 2012. **3**(2).

2. Boles, B.R., et al., *Identification of genes involved in polysaccharide-independent Staphylococcus aureus biofilm formation.* PLoS One, 2010. **5**(4): p. e10146.

3. Schilcher, K., et al., *Processing, Export, and Identification of Novel Linear Peptides from Staphylococcus aureus.* mBio, 2020. **11**(2).

4. Baba, T., et al., *Genome and virulence determinants of high virulence community-acquired MRSA.* Lancet, 2002. **359**(9320): p. 1819-27.

5. Flack, C.E., et al., *Differential regulation of staphylococcal virulence by the sensor kinase SaeS in response to neutrophil-derived stimuli.* Proc Natl Acad Sci U S A, 2014. **111**(19): p. E2037-45.

6. Clewell, D.B., et al., *Streptococcus faecalis sex pheromone (cAM373) also produced by Staphylococcus aureus and identification of a conjugative transposon (Tn918).* J Bacteriol, 1985. **162**(3): p. 1212-20.

7. Bose, J.L., P.D. Fey, and K.W. Bayles, *Genetic tools to enhance the study of gene function and regulation in Staphylococcus aureus.* Appl Environ Microbiol, 2013. **79**(7): p. 2218-24.

8. Lauderdale, K.J., et al., *Biofilm dispersal of community-associated methicillin-resistant Staphylococcus aureus on orthopedic implant material.* J Orthop Res, 2010. **28**(1): p. 55-61.

9. Pang, Y.Y., et al., *agr-Dependent interactions of Staphylococcus aureus USA300 with human polymorphonuclear neutrophils.* J Innate Immun, 2010. **2**(6): p. 546-59.

10. Ibberson, C.B., et al., *Hyaluronan Modulation Impacts Staphylococcus aureus Biofilm Infection.* Infect Immun, 2016. **84**(6): p. 1917-1929.
