# Supplementary material for: The Staphylococcus aureus CamS lipoprotein is a repressor of toxin production that shapes host-pathogen interaction
Source: PLoS Biol. 2024 Jan 5;22(1):e3002451. doi: 10.1371/journal.pbio.3002451 (PMC10769083; doi:10.1371/journal.pbio.3002451)
Supplement: S4 Table — (DOCX) [file pbio.3002451.s007.docx]

**S4 Table: Primers used in this study**

| SEQUENCES | SOURCE | IDENTIFIER |
| --- | --- | --- |
| **Primers** (5’ – 3’ sequence, RE-sites underlined, nt for AA exchange in bold) | | |
| GAAAGGTACCATTGCTGGTCA | This Paper | KAS284 |
| CCGGGTTCCAAGAATCTCTA | This Paper | KAS285 |
| GCAACGACTCAAGCAAATTC | This Paper | KAS408 |
| TCGGCAATTGGTCTAATATTTC | This Paper | KAS409 |
| CCAAGGTGCAGAAATCATCA | This Paper | KAS432 |
| CTGGACCAGTTGGGTCTTGT | This Paper | KAS433 |
| ACGTCGTTGATTATGCACCA | This Paper | KAS434 |
| TTAGCGCCATCTTGTCTGTG | This Paper | KAS435 |
| CCTCTTGCCAATCCGTTATT | This Paper | KAS436 |
| ATCCATTACCACCGAGTGATG | This Paper | KAS437 |
| CGGAATTCTTAAGTATTGGACGAACAGGTG | (1) | KAS26 |
| CCGCTCGAGttaCTTCATCGACATACCCCTCT | (1) | KAS27 |
| CCGCTCGAGCCGCAAGTTCATATTTACAGT | (1) | KAS28 |
| ACGCGTCGACGCGATGGTTATGGTCCCTAT | (1) | KAS29 |
| TGCCACCTGACGTCTAAGAA | (1) | KAS169 |
| CCTCACATTTGTGCCACCTA | (1) | KAS170 |
| GGTGCATCGTTCAGTCCAC | (1) | KAS41 |
| AACGGTAACTGAAGCGGAAT | (1) | KAS42 |
| TCAACAGGATGAGATGGGATT | (1) | KAS33 |
| TCTGGTTTCGTATCTGGTGGT | (1) | KAS34 |
| GTTGTTGTCGACAGACAGAATGGGGCAGA | This Paper | KAS266 |
| CCCCGAATTCAGCTATACTCGAGTTATGACTAACATTGGTACCTTTT | This Paper | KAS281 |
| GTTGTTGAATTCTTATGAGCACGATTTCTTCA | This Paper | KAS268 |
| GTTGTTCTCGAGTTTTGATTAACATTAGTACCTTTTT | This Paper | KAS269 |
| GTTGTTGAATTCTCATATGCCAACCCATTGTC | This Paper | KAS270 |
| CCCCGTCGACTCCTATAGCTAGCAAGTCCTCTTGCCTGGCTTT | This Paper | KAS282 |
| CCCCGAATTCAGCTATAGCTAGCCCGCAAGTTCATATTTACAGTAATT | This Paper | KAS283 |
| GTTGTTGTCGACGCGATGGTTATGGTCCCTAT | This Paper | KAS272 |
| GTTGTTGAATTCAGCTGCAATTATGAGCACGA | This Paper | KAS192 |
| TGTTGTTGTCGACAGCTGCCATTCAACAAGCTAA | This Paper | KAS193 |
| ATTACAGCTATC**GCTGTA**CTCGCTGCTTGTGGTAACCATAAGGATGA | This Paper | KAS194 |
| AG**TACAGC**GATAGCTGTAATCAATAATACTAATGTACGCTTCATCGACATACC | This Paper | KAS195 |
| ATTACAGCTATCTTTATA**GTA**GCTGCTTGTGGTAACCATAAGGATGA | This Paper | KAS274 |
| GC**TAC**TATAAAGATAGCTGTAATCAATAATACTAATGTACGCTTCATCGACATACC | This Paper | KAS275 |
| GTTGTTGGTACCCATTTTGCTTATCTACAAATTGTTG | This Paper | KAS175 |
| GTTGTTGAGCTCCAAAGTTCGTGACATCGTTAGAG | This Paper | KAS83 |
| CCCCTCTAGAAGAAAATGAAGATATTCGTGCTTTA | This Paper | KAS44 |
| CTGCTGCTGTTGAAGAAGGTAA | This Paper | KAS65 |
| GTTGTTTAAGCTTAAGATCACCGGT | This Paper | CLM429 |
| GTTGTTGGTACCTTTCGTTCTAATAAC | This Paper | CLM430 |
| GTTGTTGCTAGCCTTAAGATCACCGGTGGCG | This Paper | CLM463 |
| GTTGTTGAATTCTTAGTGGTGGTGGTG | This Paper | CLM332 |
| CCCCTCTAGACGGTGGCGGTAATAACTACACT | This Paper | KAS450 |
| CCCGGTACCGATTTGAGGAAACAATAATCAA | This Paper | KAS451 |
| GTTGTTTCTAGAAGTATCAACGATCTTATTAACG | This Paper | KAS418 |
| GTTGTTGGTACCGTTGATATGTATCGACATGTG | This Paper | KAS419 |
| CCCCTCTAGACGTTGATTCTCAATCGAATCT | This Paper | KAS446 |
| CCCCGGTACCATATATATAATAATCCATTTGTAAGC | This Paper | KAS447 |
| CCCCTCTAGACAATCGGTTACCTTAAATTGTTTAC | This Paper | KAS448 |
| CCCCGGTACCAATAATAATATATTTTGGGCGTTA | This Paper | KAS449 |
| GAGTCAGTGAGCGAGGAAGC | This Paper | KAS113 |
| GGAAACAAAAAGAGTATTTAGCTAGTG | This Paper | KAS116 |

Reference:

1. Schilcher K, Caesar LK, Cech NB, Horswill AR. Processing, Export, and Identification of Novel Linear Peptides from Staphylococcus aureus. mBio. 2020;11(2).
